# Evaluation of variant calling algorithms for wastewater-based epidemiology using mixed populations of SARS-CoV-2 variants in synthetic and wastewater samples

**DOI:** 10.1099/mgen.0.000933

**Published:** 2023-04-19

**Authors:** Irene Bassano, Vinoy K. Ramachandran, Mohammad S. Khalifa, Chris J. Lilley, Mathew R. Brown, Ronny van Aerle, Hubert Denise, William Rowe, Airey George, Edward Cairns, Claudia Wierzbicki, Natalie D. Pickwell, Matthew Carlile, Nadine Holmes, Alexander Payne, Matthew Loose, Terry A. Burke, Steve Paterson, Matthew J. Wade, Jasmine M. S. Grimsley

**Affiliations:** ^1^​ Analytics & Data Science Directorate, UK Health Security Agency, London SW1P 3JR, UK; ^2^​ Department of Infectious Disease, Imperial College London, London SW7 2AZ, UK; ^3^​ Division of Biosciences, College of Health, Medicine and Life Sciences, Brunel University, London UB8 3PH, UK; ^4^​ School of Engineering, Newcastle University, Newcastle-upon-Tyne NE1 7RU, UK; ^5^​ International Centre of Excellence for Aquatic Animal Health, Centre for Environment, Fisheries and Aquaculture Science (Cefas), Clyst Honiton EX5 2FN, UK; ^6^​ Centre for Genomic Research and NERC Environmental Omics Facility, Institute of Infection, Veterinary and Ecological Sciences (IVES), University of Liverpool, Liverpool L69 7ZB, UK; ^7^​ DeepSeq, Centre for Genetics and Genomics, University of Nottingham, Queen’s Medical Centre, Nottingham NG7 2UH, UK; ^8^​ NERC Environmental Omics Facility, Ecology and Evolutionary Biology, School of Biosciences, University of Sheffield, Sheffield S10 2TN, UK

**Keywords:** SARS-CoV-2, sequencing, variant callers, VOC, wastewater

## Abstract

Wastewater-based epidemiology has been used extensively throughout the COVID-19 (coronavirus disease 19) pandemic to detect and monitor the spread and prevalence of SARS-CoV-2 (severe acute respiratory syndrome coronavirus 2) and its variants. It has proven an excellent, complementary tool to clinical sequencing, supporting the insights gained and helping to make informed public-health decisions. Consequently, many groups globally have developed bioinformatics pipelines to analyse sequencing data from wastewater. Accurate calling of mutations is critical in this process and in the assignment of circulating variants; yet, to date, the performance of variant-calling algorithms in wastewater samples has not been investigated. To address this, we compared the performance of six variant callers (VarScan, iVar, GATK, FreeBayes, LoFreq and BCFtools), used widely in bioinformatics pipelines, on 19 synthetic samples with known ratios of three different SARS-CoV-2 variants of concern (VOCs) (Alpha, Beta and Delta), as well as 13 wastewater samples collected in London between the 15th and 18th December 2021. We used the fundamental parameters of recall (sensitivity) and precision (specificity) to confirm the presence of mutational profiles defining specific variants across the six variant callers. Our results show that BCFtools, FreeBayes and VarScan found the expected variants with higher precision and recall than GATK or iVar, although the latter identified more expected defining mutations than other callers. LoFreq gave the least reliable results due to the high number of false-positive mutations detected, resulting in lower precision. Similar results were obtained for both the synthetic and wastewater samples.

## Data Summary

All data used to generate figures have been deposited under ENA Accession numbers ERA14897883 and ERA14897645. Codes used to analyse the data have been uploaded on Github under https://github.com/mookhalifa/wastewater_variant_caller_comparison.git.

Mutation patterns used to describe VOCs and VUIs were taken from mutation patters were taken from https://github.com/phe-genomics/variant_definitions.

**Impact Statement**

Since the declaration of the pandemic in March 2020 by the World Health Organization (WHO), many laboratories have made substantial contributions to understanding the spread of COVID-19 (coronavirus disease 19), the biology and transmission of SARS-CoV-2 (severe acute respiratory syndrome coronavirus 2), as well as novel studies concerning environmental detection of the virus to support public-health systems around the world. In England, the Environmental Monitoring for Health Protection (EMHP) programme, part of the Department of Health and Social Care, has been monitoring SARS-CoV-2 since 2020, covering up to 75 % of the population. In this article, we have used a portion of the wealth of data collected over the last 2 years to interrogate whether wastewater samples are correctly analysed when it comes to identifying variants of the same circulating virus. While many laboratories have published excellent work in wastewater-based epidemiology (WBE), it is our understanding that a basic investigation regarding the ability of tools to efficiently identify these variants was lacking. Having worked with some of the variant callers within our group, we proposed this article, which we believe covers a good understanding of what tools can be used to analyse wastewater samples. In this work, we describe key parameters used to assess the efficacy of variant callers, namely sensitivity and specificity (also known as recall and precision), as well as comparing the frequency at which known mutations are called. There is a high significance behind these results, as we can clearly show that, using our datasets, some callers do indeed outperform others. To our knowledge, at present, no such comparison work has been done using environmental samples, and we believe this work will lay the basic foundations for future, more detailed and specific studies in WBE.

## Introduction

On March 11th 2020, the World Health Organization (WHO) declared a global pandemic following the rapid spread of a novel coronavirus, severe acute respiratory syndrome coronavirus 2 (SARS-CoV-2) [1], which causes coronavirus disease 19 (COVID-19). Since then, wastewater-based epidemiology (WBE) has proven a promising tool to detect and monitor SARS-CoV-2, act as a proxy for infections within certain regions/communities, and provide an early warning of disease outbreaks [2]. It has been widely used across the globe to complement conventional clinical surveillance, which is limited in population coverage, capacity or engagement (e.g. self-testing/reporting) [3]. In this regard, it is evident that WBE can be used to monitor disease prevalence in a community, allowing targeted public-health measures to be implemented at relative pace and geographical specificity, in combination with other data. Moreover, WBE is non-invasive and less biased than clinical data, making it a valuable molecular surveillance tool [4, 5].

Since the initial outbreak of SARS-CoV-2, several variants of concern (VOCs), variants under investigation or monitoring (VUIs/VUMs) and variants of interest (VOIs) have circulated globally. According to the WHO, as of May 2022, there have been five VOCs (Alpha, Beta, Gamma, Delta and Omicron), eight VOIs (Epsilon, Zeta, Eta, Theta, Iota, Kappa, Lambda, Mu) and two VUIs (B.1.640 and XD) [6]. While VOCs have transmitted worldwide, VUIs are country-specific, with over 200 sub-lineages of the main circulating variants reported by individual countries [7]. In England, the Horizon Scanning Programme, part of the UK Health Security Agency (UKHSA), has been monitoring circulating variants, including VOCs, VUIs/VUMs and VOIs, identified by deep sequencing of a large cohort of COVID-19 positive patients by COG-UK (COVID-19 Genomics UK) [8, 9]. Since the declaration of the pandemic, this totals over 2 million patients in the UK alone [10]. However, the sequencing datasets generated lacked asymptomatic cases and cases not sequenced.

The Environmental Monitoring for Health Protection (EMHP) programme, part of the UKHSA, has used reports produced by the Horizon Scanning Programme to monitor the same variants in wastewater collected in England. However, the analysis of SARS-CoV-2 sequences from wastewater samples is more complicated than clinical samples obtained using nasopharyngeal swabs. Discrepancies between clinical and wastewater samples have been observed; in particular, the mixed strain nature of wastewater samples, the more degraded nature of viral genomes and, consequently, the inability to obtain a consensus genome sequence for each of the samples analysed. These differences can be accounted for by the nature and characteristics of the samples (wastewater vs clinical) and characteristics impacting the ability to extract and analyse the samples, such as virus titre, which is considerably lower in wastewater samples, and that, in turn, may affect variant calling, such as sample preparation, with additional steps such as centrifugation and filtration methods required to purify the samples from chemicals and other sources of contamination, and platform-dependent sequencing errors [11–14].

Several bioinformatics pipelines have been developed to specifically detect SARS-CoV-2 sequences and variants in wastewater samples, including nf-core/viralrecon [15] and V-PIPE SARS-CoV-2 [16]. However, most studies have relied heavily on the artic pipeline initially designed with clinical samples in mind or, as in the case of the EMHP programme in England, an adaptation of this pipeline. Common sequencing pipelines, including artic, involve the removal of low-quality sequencing reads, followed by read mapping and variant calling to define mutations found in the sample (SNPs and INsertions/DELetions) [17]. This is performed by highly specialized tools known as variant callers. The artic pipeline for sequence analysis of clinical samples utilizes iVar, which relies on the SAMtools mpileup command as its variant calling function [17]. While this has been well documented in clinical studies, very little is known about its performance for wastewater samples. To address this knowledge gap, the EMHP programme in England adapted this protocol, using VarScan as an alternative to iVar, delivering significantly improved results when applied to wastewater samples [18, 19]. Before screening for sequence changes, VarScan uses the BAM alignment file as the input to score each of the reads produced during sequencing. If reads are found to align to multiple locations and/or are of low quality, they are automatically discarded. For the remaining reads, SNPs and Indels are compiled for each of the locations across the viral genome and validated depending on factors such as the overall coverage, the number of reads across the site of the mutation and base quality, among others.

Several genomic studies have compared and highlighted the impact that variant caller choice has on the analysis pipeline, including iVar [17], GATK [20], LoFreq [21], FreeBayes [22] and BCFtools [23]. FreeBayes is a haplotype-based variant caller where variants are called based on the sequences of reads aligned to a particular target rather than the specific alignment. One of the main advantages of this method is that it bypasses the problem of identical sequences that might align to multiple locations. However, GATK uses HaplotypeCaller as a tool to call germline SNPs and Indels via local re-assembly of haplotypes [22]. More specifically, it assembles and realigns reads to their most likely haplotypes. Comparison with the reference of choice is used to calculate the likelihood of each possible genotype and call possible variants. LoFreq is a high-quality and highly sensitive tool to detect variants in heterogeneous samples, such as tumour samples [21]. It was developed under the assumption that it is hard to distinguish true variants from sequencing errors. In this regard, LoFreq is a very robust and sensitive variant caller that uses base-called quality values to call variants accurately. It differs from other callers as it can find SNPs and Indels at a frequency below the mean sequencing error. As such, it is not ideal for low-coverage genomes. BCFtools is a collection of several commands, among which *call* is used for SNP/indel calling [23]. It generates the *mpileup* from the BAM alignment reads and then computes the variant calling. This step is the same as VarScan, which generates *mpileup* using SAMtools. iVar uses the output of the SAMtools *mpileup* command to call variants as VarScan and BCFtools; however, it is not adapted for use in mixed strain samples, such as those derived from wastewater where mixed populations are found in the same sample [17, 24, 25]. Indeed, it is globally acknowledged that the detection of defining SNPs and Indels allows the assignment of VOCs, VUIs, VUMs and VOIs to wastewater samples; thus, their accurate identification is paramount for variant detection in the context of WBE.

In this paper, we evaluated the performance of six different variant callers and their ability to detect SNPs and Indels in samples containing a mixture of synthetic SARS-CoV-2 control variants, as well as wastewater samples collected across Greater London during the pandemic.

## Methods

### Sample library preparation and sequencing

The synthetic SARS-CoV-2 control variant dataset contained samples that consisted of a mix of three VOC genomes: Alpha (Control 15), Beta (Control 16) and Delta (Control 23) synthesized by TWIST Bioscience (Table 1). For each sample in the synthetic dataset, three of these genomes were mixed in different ratios up to a total concentration of 200 genome copies l^−1^, ranging from 0 to 100 % of each synthetic genome, in quadruplicates. The mutation profile of each of the synthetic genomes is provided in Table S1 (available with the online version of this article). Wastewater samples were collected between the 15th and 18th December 2021 in eight locations across the city of London, UK. Most of these samples were found to be positive for both the Delta and Omicron lineages using the bioinformatics pipeline developed by the EMHP programme (data not shown). Wastewater samples were clarified, concentrated and RNA extracted according to the Quantification of SARS-CoV-2 in Wastewater General Protocol v1.0 (https://www.cefas.co.uk/media/offhscr0/generic-protocol-v1.pdf). Sequencing libraries (tiled amplicons) were generated using the EasySeq SARS-CoV-2 WGS library prep kit (NimaGen) and the NimaGen v3 (wastewater samples) and v4 (synthetic samples) primer schemes, following the Wastewater Sequencing using the EasySeq RC-PCR SARS-CoV-2 (NimaGen) v2.0 protocol [26]. The libraries were sequenced on an Illumina NovaSeq 6000 (2×150 bp) at the University of Liverpool sequencing centre (synthetic samples) or an Illumina NextSeq 500 (2×150 bp) at the University of Nottingham sequencing centre (wastewater samples).

### Read pre-processing, mapping, primer trimming and variant calling

The artic pipeline (ncov2019-artic-nf; Illumina workflow) [27] was used to process the raw Illumina reads. Briefly, amplicon reads were pre-processed using Trim Galore v0.6.5 [28], mapped to the reference SARS-CoV-2 genome [ENA (European Nucleotide Archive) accession no. MN908947.3, NCBI (National Center for Biotechnology Information) GenBank accession no. NC_045512.2] using bwa v0.7.17 [29], followed by primer trimming using iVar v1.3 and bed files containing the genome positions of the primers used to generate the amplicons (NimaGen v3 and v4 primer schemes for wastewater and synthetic samples, respectively). The resulting BAM files were sorted and subsequently indexed using SAMtools v1.13 [23] before analysis with six different variant callers: iVar v1.3.1, LoFreq v2.1.3.1, BCFtools v1.13, GATK HaplotypeCaller v3.8, VarScan v2.4.4 and FreeBayes v0.9.21. To avoid introducing biases across the variant callers, only parameters common to those available from VarScan were chosen. VarScan is the caller with the least number of parameters as it allows one to choose only from min-coverage (minimum read depth at a position to make a call), min-reads2 (minimum supporting reads at a position to call variants), min-avg-qual (minimum base quality at a position to count a read), min-*var*-freq (minimum variant allele frequency threshold) and *P* value (*P* value threshold for calling variants). Also, it should be noted that it is practically impossible and computationally intensive to test all the parameters from all the callers, and default parameter choices are a practical alternative that also more easily facilitate comparisons between off-the-shelf variant callers [30–32].

The artic pipeline outputs a list of mutations (SNPs and Indels) detected for each variant using iVar, but this was re-run separately after matching the common parameters across the various callers being investigated. All parameters are described in Supplementary file 1. The sorted, indexed and primer-trimmed BAM files were used directly to run variant calling with FreeBayes, iVar, BCFtools and VarScan, while LoFreq and GATK HaplotypeCaller required pre-processing of these BAM files first. Since LoFreq required indel quality information in the BAM file to process indel calls, we used the LoFreq command *indelqual* to insert quality score for each indel, based on the dindel algorithm [33]. GATK HaplotypeCaller, requires reads to be grouped (using *AddOrReplaceReadGroups* from Picard) and duplicates (*MarkDuplicatesSpark* from GATK) were marked before variant calling. All the variant callers generated outputs in variant call format (vcf) files except iVar, which reported outputs as tsv (tab-separated values) files. A Python script (ivar_variants_to_vcf.py) was used to convert the tsv file format to vcf file format [34].

### Vcf file processing, analysis and statistical methods

QuasiModo is a tool that evaluates the results of strain resolved analyses on mixed strain samples including variant calling and genome assembly [35]. It does this by taking vcf files generated from the different variant callers and two genomic reference files, the first being the reference against which samples were mapped in the BAM-file-generating process, and the second reference being a ground-truth genome known to be found in the mixed strain samples. Therefore, we evaluated the performance of the different variant callers by comparing lists of mutations identified by each of the variant callers to a second reference genome (for ground truthing). The reference SARS-CoV-2 genome sequence was downloaded from NCBI GenBank (accession no. MN908947.3) and the SARS-CoV-2 variant genomes were obtained from GISAID: Alpha (EPI_ISL_601443), Beta (EPI_ISL_678597), Delta (EPI_ISL_1544014), Omicron-England (EPI_ISL_7718520), Omicron-Hong Kong (EPI_ISL_6841980), Omicron-Australia (EPI_ISL_7190366) and Gamma (EPI_ISL_792683). Briefly, sequences from each sample were aligned to the reference genomes using MUMmer4 [27] to identify SNPs and Indels that are present in the ground-truth genome and each variant call was then categorized as either a true positive (TP), a false positive (FP) or a false negative (FN). A TP is defined as one that was found by the variant caller being tested in both the sample and the reference. A true negative is a lack of a mutation detected by the variant caller where there is no mutation present in the reference file. A FP is a mutation reported by the variant caller but not present in the original reference, while a FN is a mutation not detected by the variant caller, but that is found in the reference [35–38].

These values are used to calculate the recall and precision, also known as sensitivity and specificity, respectively:

Recall (R, fraction of truly existing variants) = TP/(TP+FN)

Precision (P, fraction of predicted true variants) = TP/(TP+FP).

In addition, once recall and precision are calculated, a ratio of the two can be derived, known as the F1 score [35]:

F1 = 2×(P×R)/(P+R).

For the synthetic control samples, 455 vcf files [generated for 19 synthetic samples (quadruplicates) from six variant callers, with one failed replicate for GATK] were analysed using the MN908947.3 reference file as the mapping genome and each of the SARS-CoV-2 VOC reference genomes (Alpha, Beta, Delta and Gamma). Similarly, for wastewater samples, we generated 77 vcf files (from 13 unique samples among six variant callers, with one failed sample for GATK), using the MN908947.3 reference and each of the SARS-CoV-2 VOC genomes [Alpha, Beta and Delta, Omicron (Hong Kong), Omicron (Australia) and Omicron (England), and Gamma; Table 1]. The Gamma (P1) variant reference file (Table 1) served as a negative control in our bioinformatics analysis, as it was not included in the synthetic mixtures nor found in the wastewater samples. We adapted the method described by Deng *et al*. [35] to generate a table with calculated values for each of the vcf files, from which recall and precision were plotted using R v4.1.3 and ggplot2 [39]. Output from all the vcf files was used by *vcfstats* from the vcflib package [40] to generate variant statistics for each of the vcf files. *vcfstats* generates a two-column output for each vcf file, with counts for SNPs, MNPs (multiple nucleotide polymorphisms), Indels and various other parameters. The numbers of SNPs, Indels and MNPs for each vcf file were plotted using RStudio, ggplot2 package [39]. Frequencies of the defining mutations for each of the variant genomes were extracted from the vcf files using BCFtools [23] and plotted using RStudio, ggplot2 [39].

**Table 1.** List of synthetic genomes synthesized by TWIST Bioscience used for the comparison test Defining mutation patterns were taken from https://github.com/phe-genomics/variant_definitions. MNV = Multiple nucleotide variation.

TWIST part no.	GISAID ID	GISAID name	PANGO lineage	WHO label	Defining mutation	Total no. of mutations in TWIST genome	SNPs	Indels	MNVs
103909	EPI_ISL_601443	England/MILK9E05B3/2020	B.1.1.7	Alpha	15	28	22	4	2
104043	EPI_ISL_678597	South Africa/KRISPEC-K005299/2020	B.1.351	Beta	15	25	23	2	0
104533	EPI_ISL_1544014	India/MH-NCCS P1162000182735/2021	B.1.617.2	Delta	13	37	30	7	0
104044	EPI_ISL_792683	Japan/IC-0564/2021	P.1	Gamma	23	24	21	3	0
105204	EPI_ISL_6841980	Hong Kong/HKU-211129-001/2021	B.1.1.529 BA.1	Omicron	17	59	45	14	0
105345	EPI_ISL_7190366	Australia/QLD2568/202	B.1.1.529 BA.2	Omicron	20	57	48	9	0
105346	EPI_ISL_7718520	England/MILK-2DF642C/2021	B.1.1.529 BA.2	Omicron	20	58	48	9	0

To test whether the distribution of precision, recall and F1 scores for each variant caller was significantly different from another, we applied the Kruskal–Wallis one-way ANOVA test using the Python SciPy package v1.9.1 (https://docs.scipy.org/doc/scipy/reference/generated/scipy.stats.kruskal.html). Following this, a post hoc Dunn test using scikit-posthocs package v0.7.0 (PyPI – https://scikit-posthocs.readthedocs.io/en/latest/generated/scikit_posthocs.posthoc_dunn/) was performed to evaluate the pairwise differences between callers. These tests were performed separately on the synthetic samples, the wastewater samples and both sets of samples together, for each score. Each set of quadruplicate synthetic samples were aggregated by median score before applying the relevant tests.

## Results

### Sensitivity and specificity of six variant callers across mixed synthetic genome samples

We ran VarScan, GATK, iVar, FreeBayes, LoFreq and BCFtools across 19 mixed ratio synthetic samples, in quadruplicates. Basic sequencing statistics for all the samples calculating recall, precision and F1 score values are summarized in Fig. S1(a).

**Fig. 1.**
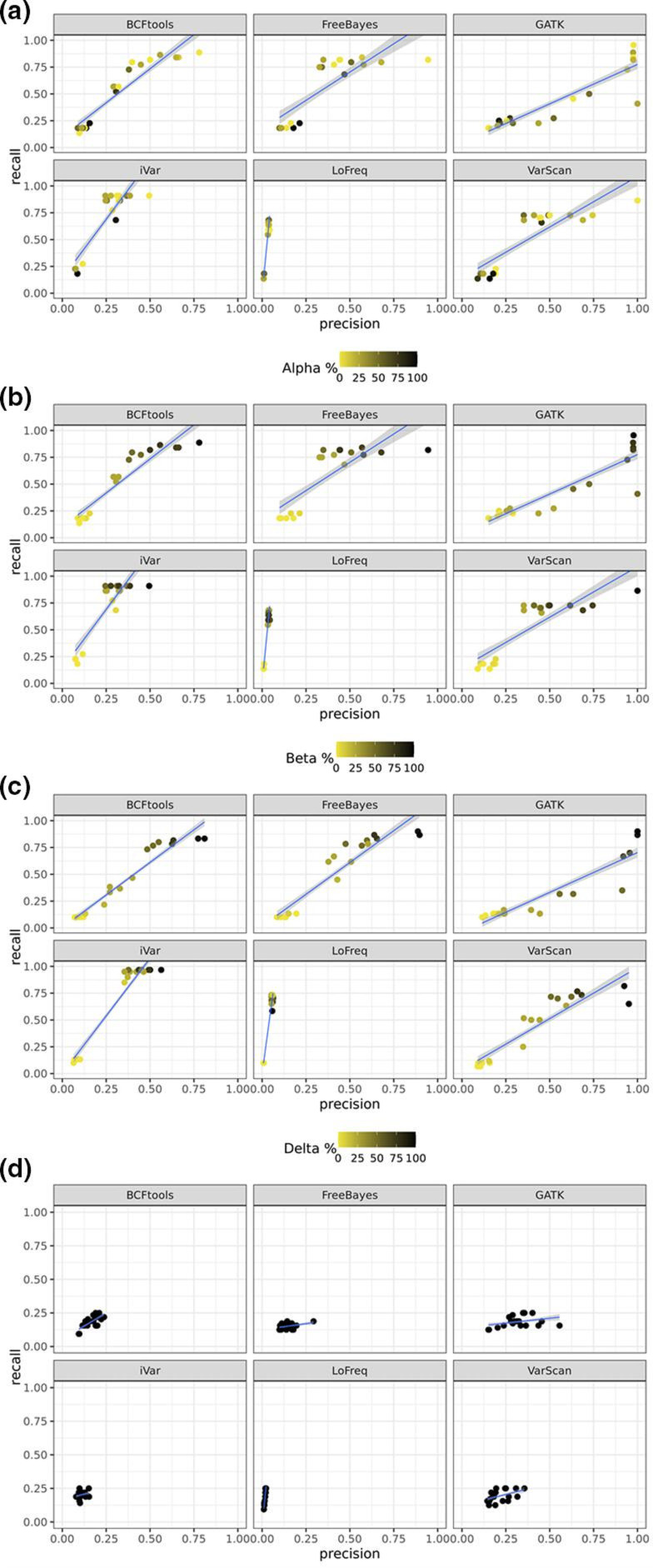
Point plots of precision versus recall for synthetic samples, grouped and faceted by variant caller, coloured by percentage present in the mix (yellow, 0 %; black, 100 %). A linear regression for each plot is also present. (a) Comparison of the synthetic samples to the Alpha VOC reference, (b) the Beta VOC reference, (c) the Delta VOC reference and (d) Gamma VOC reference. As there was no mixed ratio for the Gamma VOC that we have used as a negative control, no gradient was applied. Except for LoFreq and iVar, all the callers show a high precision and recall, and this is proportional to the percentage of the VOC in the mix: indeed, samples with a high percentage of a VOC (e.g. close to 100 %, in black) tend to have a higher precision and recall, compared to those samples that have a lower percentage of the VOC being plotted (e.g. closer to 0 %, in yellow).

We calculated recall and precision for each variant within the samples and plotted these separately for each variant caller (Figs 1a–d and S2a–d), by also highlighting the percentage of each variant in the mixed sample as described in Table 2. Given that all four replicates yielded very similar results (reliable technical replicates), we ran the median of these for all the synthetic samples plots. As shown in Fig. S3, we picked three samples that only contained 100 % of one specific VOC in the mix, namely Alpha, Beta or Delta (shown in Table 2 marked by *) to show the validity of the replicates. Indeed they all had a similar distribution of the four replicates.

**Table 2.** Mixed synthetic samples used to compare the six variant callers The samples marked with * were also used for comparison of technical replication.

No.	Sample mix name	Synthetic (VOC) genome in the sample	Synthetic genome ratio (%) in the mix
1*	100_Alpha|0_Beta|0_Delta	Alpha	100/0/0
2*	0_Alpha|100_Beta|0_Delta	Beta	0/100/0
3*	0_Alpha|0_Beta|100_Delta	Delta	0/0/100
4	25_Alpha|75_Beta|0_Delta	Alpha/Beta	25/75/0
5	50_Alpha|50_Beta|0_Delta	Alpha/Beta	50/50/0
6	75_Alpha|25_Beta|0_Delta	Alpha/Beta	75/25/0
7	95_Alpha|5_Beta|0_Delta	Alpha/Beta	95/5/0
8	10_Alpha|70_Beta|20_Delta	Alpha/Beta/Delta	10/70/20
9	20_Alpha|70_Beta|10_Delta	Alpha/Beta/Delta	20/70/10
10	25_Alpha|25_Beta|50_Delta	Alpha/Beta/Delta	25/25/50
11	25_Alpha|50_Beta|25_Delta	Alpha/Beta/Delta	25/50/25
12	50_Alpha|25_Beta|25_Delta	Alpha/Beta/Delta	50/25/25
13	50_Alpha|0_Beta|50_Delta	Alpha/Delta	50/0/50
14	25_Alpha|0_Beta|75_Delta	Alpha/Delta	25/0/75
15	75_Alpha|0_Beta|25_Delta	Alpha/Delta	75/0/25
16	0_Alpha|25_Beta|75_Delta	Beta/Delta	0/25/75
17	0_Alpha|5_Beta|95_Delta	Beta/Delta	0/5/95
18	0_Alpha|50_Beta|50_Delta	Beta/Delta	0/50/50
19	0_Alpha|75_Beta|25_Delta	Beta/Delta	0/75/25

Our results show that all the variant callers correctly identified each SARS-CoV-2 variant in the synthetic mixes. At the time of writing, the tools we used to evaluate the presence of variants in a mixed sample via evaluation of their mutational profile could not be applied for mixed samples containing more than two variants or strains; therefore, we investigated the correct identification of the percentage by analysing the variants independently rather than confirming that all variants were found simultaneously in the same sample. Our results suggest that, in general, the greater the proportion of a variant (close to 100 %), the greater the chance it was called correctly (Fig. 1). Indeed, VarScan, BCFtools and FreeBayes correctly called the increased ratio of Alpha compared to the remaining variants in the mix, while iVar and LoFreq had a trend line where the increased concentration of the variants could not be observed as clearly as in the other callers, showing instead a lower precision and for the latter also a low recall (Figs S1a and S2a–c). As expected, our negative control P1 (Gamma) (Fig. 1d) did not yield any significant results, with all samples having a very low precision and recall for every caller assessed. Specifically, we observed that samples with a ratio close to zero, thus, with low concentration of a variant, tended to cluster together with low recall and low precision. Those with a higher ratio for a variant and, therefore, with more mutations to be detected across several variants, are distributed across the plot to reflect the increased recall, and for some callers, higher precision. Based on this initial observation, BCFtools, VarScan and Freebayes had the highest precision, followed by iVar and GATK. In addition, iVar had the highest recall for each of the variants being assessed independently, via count of their TPs, FPs and FNs compared to the other callers. This was supported by a Dunn’s test to compare the synthetic samples’ precision, recall and F1 scores of each variant caller. It confirmed that the differences in precision and F1 scores for LoFreq were significantly different to the other callers (*P*<0.01) and iVar performed best for recall and the Dunn’s test again confirmed this as statistically significant (*P*<0.01; Fig. S1a).

### Sensitivity and specificity of six variant callers across wastewater samples from London

**Fig. 2.**
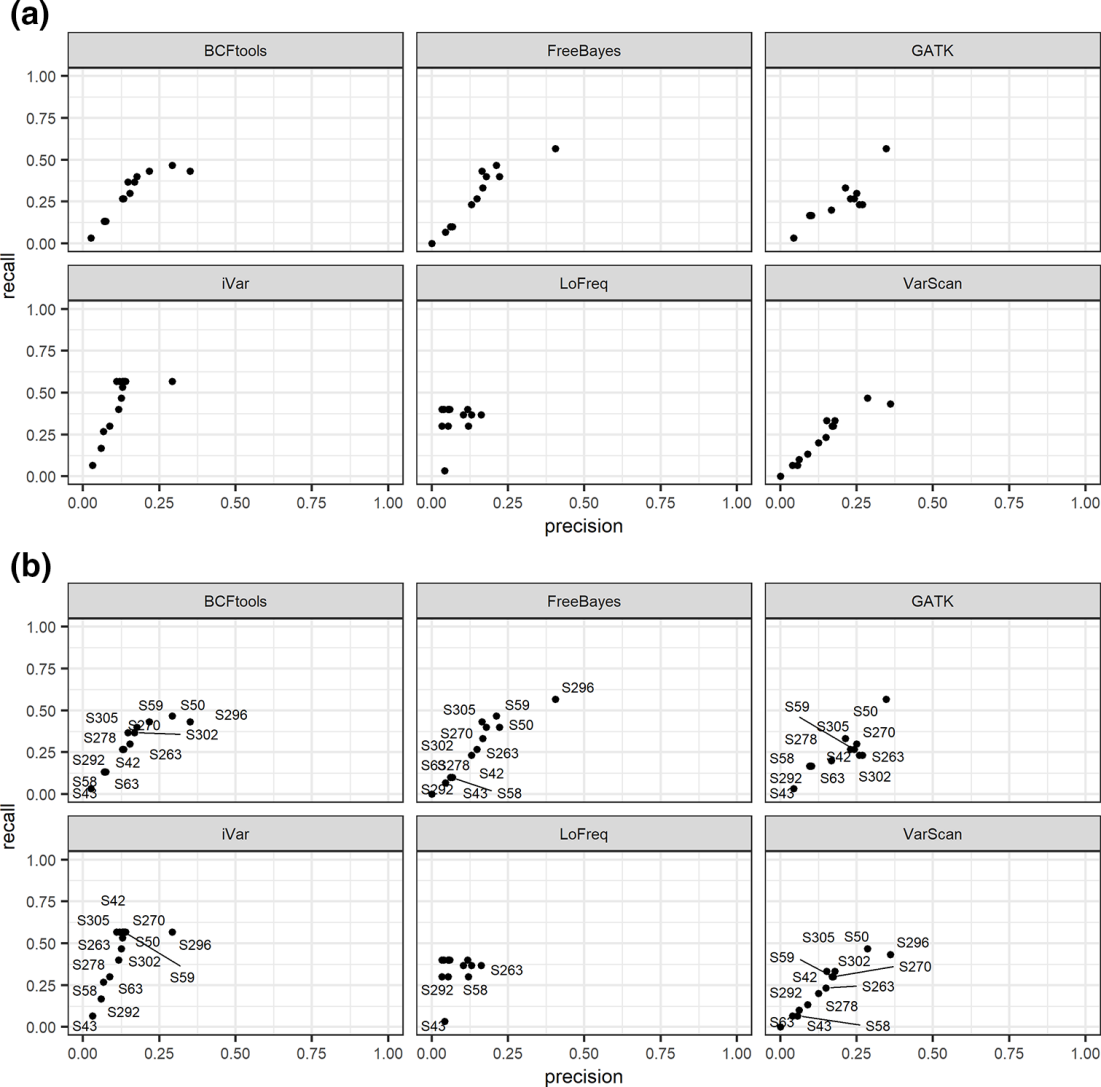
Point plots of precision versus recall for wastewater samples for the Delta VOC, faceted by variant caller. (a) Comparison of real wastewater samples to the Delta variant reference and (b) with labelled samples to better identify which samples had low recall and low precision (thus, not containing any Delta VOC). As shown in Fig. 4(c), some samples such as S296 and S50 are those containing a Delta VOC in the mix, compared to S43 seen to be negative for all the variant callers. As shown previously for the synthetic data, LoFreq has the lowest recall and precision.

We used wastewater samples collected between the 15th and 18th December 2021 from Greater London to assess whether the variant callers could identify the mutations with similar precision and recall as observed with the synthetic samples. Table 3 shows the list of the samples, dates and predicted variants known to be found in those samples, and Fig. S1(b) shows basic sequencing statistics. Samples were predicted to contain a mix of the Omicron and/or Delta and/or AY.4.2 variants [EMHP analysis based on PHE variant definition, data not shown]. Given the genome similarity between the Delta and AY.4.2 variants, we only carried out our analysis on the Delta variant mutations. Fig. 2(a, b) shows that the variant callers recognized mutations that could be identified as Delta variant for some of the samples, which indeed show a higher precision and recall compared to others, while in Fig. S3(a, b, d) we show that all three VOCs are not detected, as expected, since these were not expected to be found in the samples, compared to Delta that is shown to be found in some of the samples (Fig. S3c). Indeed, when testing for Delta variant presence, we noticed a slight increase in the precision and recall for some of those samples, which suggests that those did contain SNPs and/or Indels that could be identified as being part of the Delta variant, namely S50 and S296, although the latter was not called by GATK. Consistent with the data in Table 3, some of the samples were found not to contain a Delta variant, such as sample S43, which indeed showed a low precision and recall for all the callers, while other samples with slightly higher values reflected that they did contain a mix of both Omicron and Delta (samples S58, S292, S63). As shown for the synthetic samples, LoFreq was the only variant caller that called with the lowest precision for all the samples analysed, followed by iVar, while recall values for LoFreq were sparser, yet higher than the other callers.

**Table 3.** List of wastewater samples collected across London between the 15th and 18th December 2021 Samples in bold were also used to generate UpSet plots for Fig. 5.

No.	Wastewater sample	Sample code	Sample collection date	VOC detected by EMHP
1	London 1	S59	15/12/2021	Delta/Omicron
**2**	**London 2**	**S42**	16/12/2021	Delta/Omicron
**3**	**London 3**	**S63**	16/12/2021	Delta/Omicron
4	London 4	S50	16/12/2021	Delta/Omicron
**5**	**London 5**	**S58**	16/12/2021	Delta/Omicron
**6**	**London 6**	**S43**	16/12/2021	Omicron
**7**	**London 7**	**S296**	18/12/2021	Delta
**8**	**London 8**	**S263**	18/12/2021	Delta/Omicron
9	London 9	S302	18/12/2021	Delta/Omicron
**10**	**London 10**	**S292**	18/12/2021	Delta/Omicron
11	London 11	S278	18/12/2021	Omicron
12	London 12	S305	18/12/2021	Omicron/AY.4.2
13	London 13	S270	18/12/2021	Omicron/AY.4.2

**Fig. 3.**
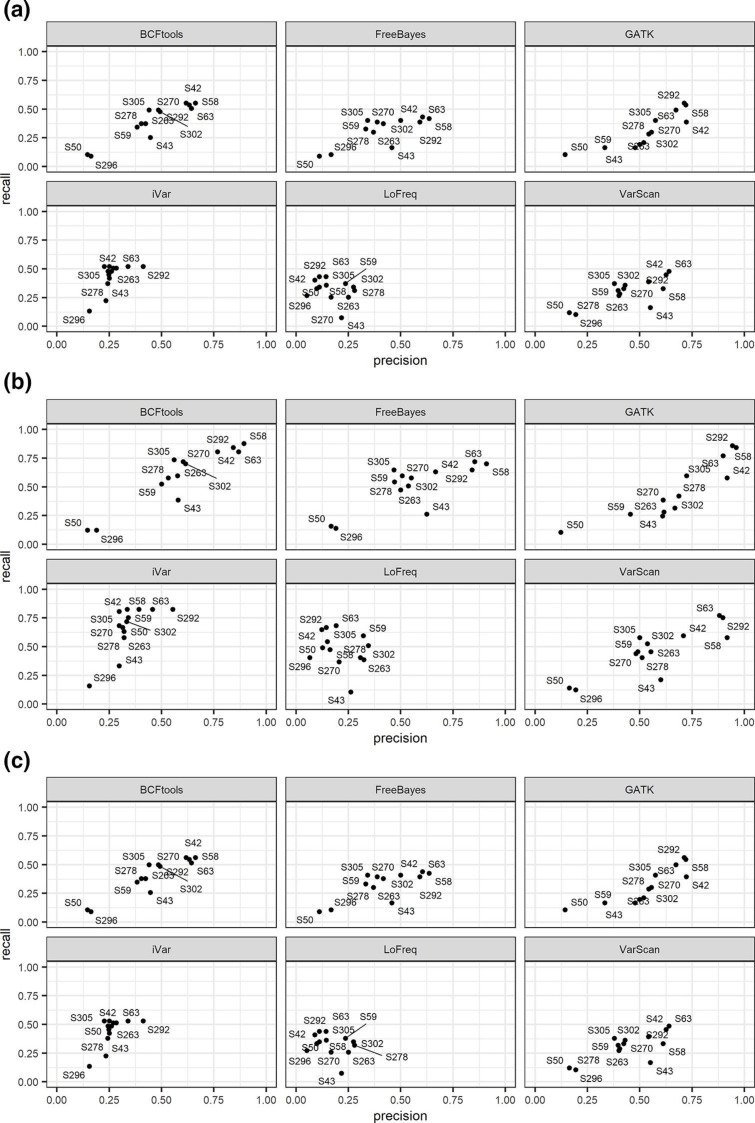
Point plots of precision versus recall for real wastewater samples for the three Omicron VOCs, faceted by variant caller: (a) Omicron England VOC reference; (b) Omicron Hong Kong VOC reference; (c) Omicron Australia VOC reference. Since the wastewater samples are known to contain the Omicron VOC, samples do show a higher precision and recall compared to the negative controls used to generate Fig. 4(a–d). Samples S63, S58, S42 and S292 had the highest precision and recall for BCFtools, Freebayes, GATK, iVar and VarScan, while LoFreq did not call with the same efficiency. S50 and S296 had the lowest precision and recall for all the callers. Notably, GATK did not call S296.

Similarly, we tested the wastewater samples for the presence of the Omicron lineage (Fig. 3a–c). We used three different references representative of this variant, namely, England, Hong Kong and Australia. As shown in Figs 3(a–c) and S5(a–c), all three variants under analysis were found in our wastewater samples and at a higher level than the one at which the Delta variant was detected for specific samples expected to have either or both variants. The degree to which each variant caller recognizes the mutations varied, with LoFreq again returning the lowest recall and precision values compared to the other callers. This was consistent with the results obtained with the synthetic data. Based on the predicted detection indicated in Table 3, the two samples identified to contain a Delta-based mutational profile as described above (S50 and S296) have now a low precision and recall when tested against any of the Omicron lineages, suggesting that in those samples we can predict to find a Delta variant rather than an Omicron. This was confirmed consistently for all the callers, although we also observed again that LoFreq did have low values as shown in the other plots, and that GATK did not call S296. As for the synthetic samples, a Dunn’s test of the pairwise scores confirmed that in terms of precision, GATK, VarScan and Freebayes were not significantly different from one other. However, the Dunn’s test on recall showed iVar to be stochastically dominant. For the combined F1 score, the same test showed that only LoFreq was significantly different from each of the other tools (*P*<0.01; Fig. S1b).

**Fig. 4.**
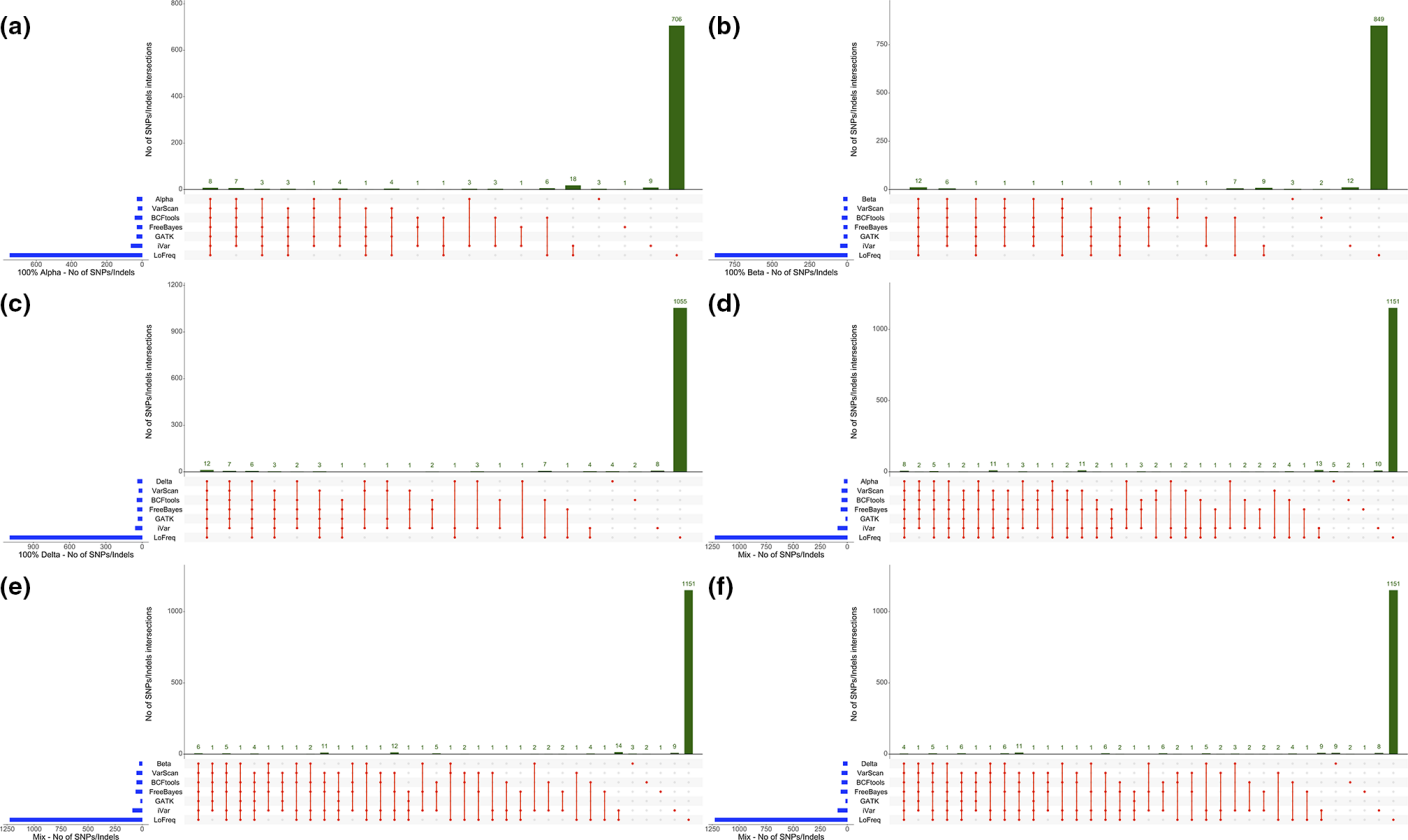
UpSet plots showing the common set of mutations found in (**a**) the Alpha reference genome and sample containing 100 % Alpha synthetic genome, (**b**) the Beta reference genome and sample containing 100 % Beta synthetic genome, (**c**) the Delta reference genome and sample containing 100 % Delta synthetic genome. (**d–f**) UpSet plots showing common mutations between mixed sample 12 (50 % Alpha, 25 % Beta and 25 % Delta synthetic genome) and (**d**) the Alpha reference genome, (**e**) the Beta reference genome and (**f**) the Delta reference genome, for six different variant callers. For each plot in (a–f), we added the VOC being tested at the top of the variant callers. Each mutation called by variant callers below the VOCs can then be compared to look at how many of the VOC-defining mutations are found by the variant caller. The figures show that not all the defining mutations are found by each of the callers, and the additional mutations each variant caller has found. Among all the callers, LoFreq is the caller with the highest number of mutations detected and much fewer corresponding to the VOC-defining list as shown by the VOC on top of the callers.

### Comparison of known variant-defining mutations found in synthetic and wastewater samples across the six variant callers

**Fig. 5.**
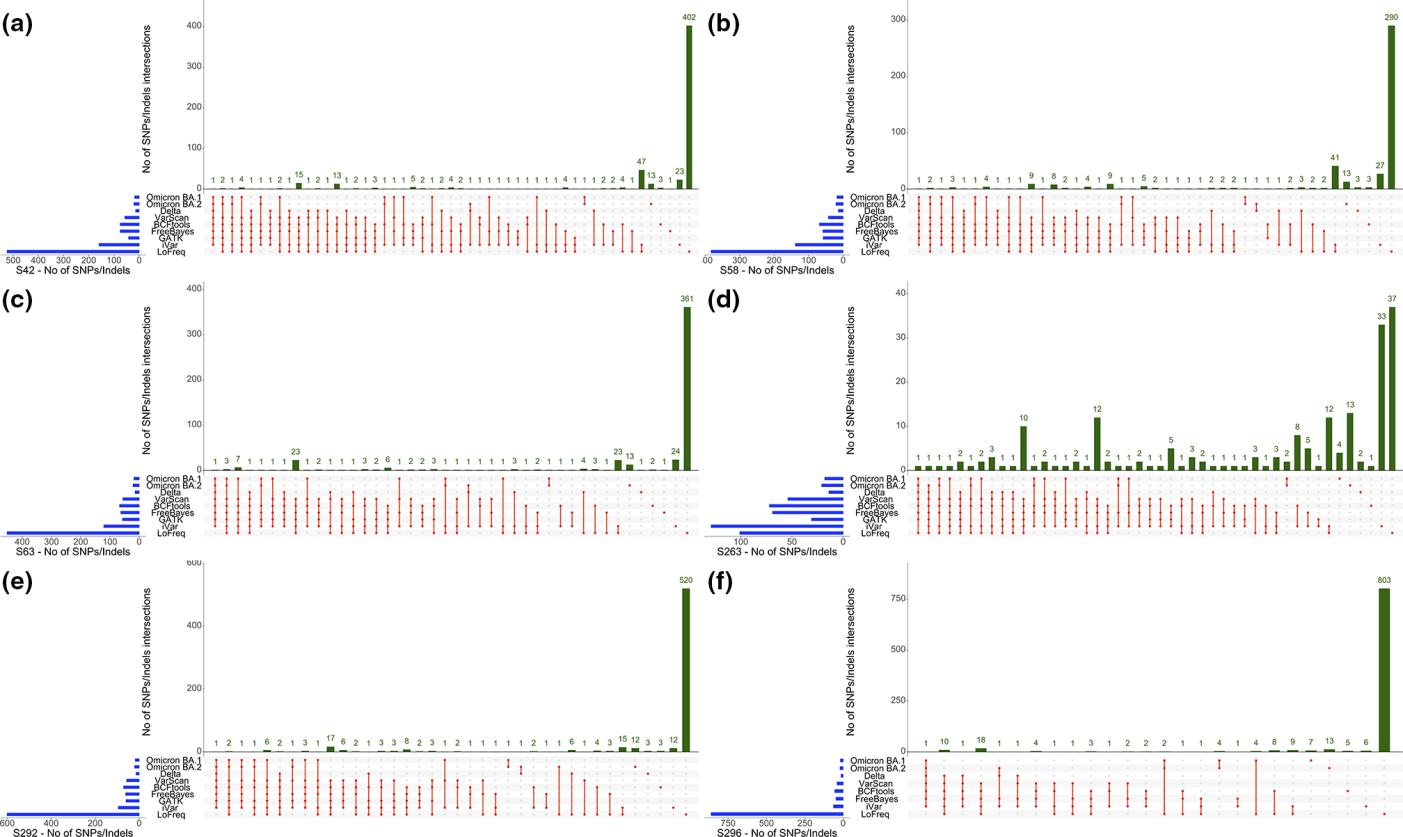
(a–f) UpSet plots of wastewater samples showing the common set of defining mutations found between Delta, Omicron BA.1 and Omicron BA.2 and wastewater samples (**a**) S42, (**b**) S58, (**c**) S63, (**d**) S263, (**e**) S292 – GATK failed and (**f**) S296, for six different variant callers. As described for Fig. 4, each mutation called by variant callers listed below Omicron BA.1, Omicron BA.2 and Delta can then be compared to look at how many of the VOC-defining mutations are found by the variant caller. The figure shows that not all the defining mutations are found by each of the callers, and the additional mutations each variant caller has found. As seen in the synthetic samples, LoFreq is the caller with the highest number of mutations detected and much fewer corresponding to the VOC-defining list as shown by the VOC on top of the callers. All callers also show the presence of unique mutations not found by the other callers and not present in the list of the defining ones as seen under Omicron BA.1, Omicron BA.2 and Delta.

We calculated the total number of known SNPs, Indels and MNPs as described by TWIST Bioscience (synthetic samples) or the PHE (wastewater samples) for each variant and compared these with those found in our synthetic (Figs 4a–f and S6a–f, Supplementary file 2) or wastewater samples (Figs 5a–f and S6a–f, Supplementary file 2) by each of the callers. SNPs/Indels bar plots were also shown in the absence of LoFreq to show the divergence among all the callers on a different scale, as shown in Fig. S7. In Figs 4(a–f) and 5(a–f), we used UpSet plots to show TPs for a subset of both synthetic and wastewater samples, respectively. For the synthetic samples, we chose the three samples with 100 % of a variant and one with a mix of the three (sample 12; Table 2). Among the wastewater samples, we chose six samples, representative of both Omicron lineages and Delta variants (Table 3, samples highlighted in bold). As shown in Fig. 4(a–f), and in concordance with the data presented above, LoFreq did call a much higher number of mutations compared to all the other variant callers in all the synthetic samples analysed, leading to a high number of FNs. When looking in more detail at how many of those were the defining mutations for each of the reference genomes (Table 1), we found that all callers identified the majority of the expected mutations for the variants being investigated, except LoFreq which only found 11/28 mutations for Alpha, 14/25 for Beta and 20/37 for Delta (Fig. 4a–c, Supplementary file 2). In addition, a set of three mutations was not detected by any of the variant callers for Alpha and Beta, and four mutations for the Delta VOCs. The detailed numbers of defining expected mutations for all the callers are described in Supplementary file 2.

For the synthetic mixed strain sample (Table 2, sample 12), we tested the presence of the defining mutations for the three VOCs of interest (Fig. 4d–f, Supplementary file 2), which were mixed in a 50 :25 : 25 ratio. As summarized in Supplementary file 2, GATK called the lowest number of expected mutations for all the three variants, followed by VarScan and LoFreq. However, we found that iVar, FreeBayes and BCFtools were the callers with the highest number of expected mutations for all three variants profiled. Five mutations for the Alpha, three for the Beta and eight for Delta were not detected by any of the callers.

Similarly, we called VOCs for the wastewater samples (in bold Table 3, Figs 5a–f and S6b, d, f, Supplementary file 2) and observed the same pattern. More specifically, we investigated how many of the Delta, Omicron BA.1 and BA.2 defining mutations were detected by each of the variant callers across the samples and compared the numbers. As shown in Fig. 5(a–f), we found that, of all the mutations detected in the wastewater samples, 13/20 mutations for BA.2 were not detected by any of the variant callers (Supplementary file 2), yielding to a very low number of defining mutations found by each caller (up to 6 total mutations). For BA.1, which has a total of 17 defining mutations, LoFreq called the least number of expected mutations for sample S58 (7/17) and S263 (6/17). Overall, all the callers found between 6 to 16 defining mutations across the samples, with iVar having the highest number of expected mutations compared to the other callers: it detected all the 16/17 mutations for BA.1 in all samples, except 10/17 for S263 and 0/17 for S296. When looking at the Delta VOC defining mutations, as observed for the other variants, there is a degree of difference within the same sample, e.g. iVar and LoFreq called 8/17 and 9/17 defining mutations, respectively, for sample S63, but all the others only 3/17. Overall, iVar seemed to have performed well for the Delta VOC, where it called the highest number of expected mutations (Supplementary file 2).

Similarly, bar plots showing the number of total SNPs, Indels and MNPs across the samples for the six variant callers were also calculated. This is shown in Fig. S6(a–f), for both synthetic and the real wastewater samples, and in absence of LoFreq in Fig. S7. Interestingly, not all the variant callers were able to recognize MNPs. As shown in Fig. S6(e, f), only FreeBayes and iVar found this type of mutations across both synthetic and real wastewater samples.

### Comparison of alternate allele frequencies across the six variant callers

**Fig. 6.**
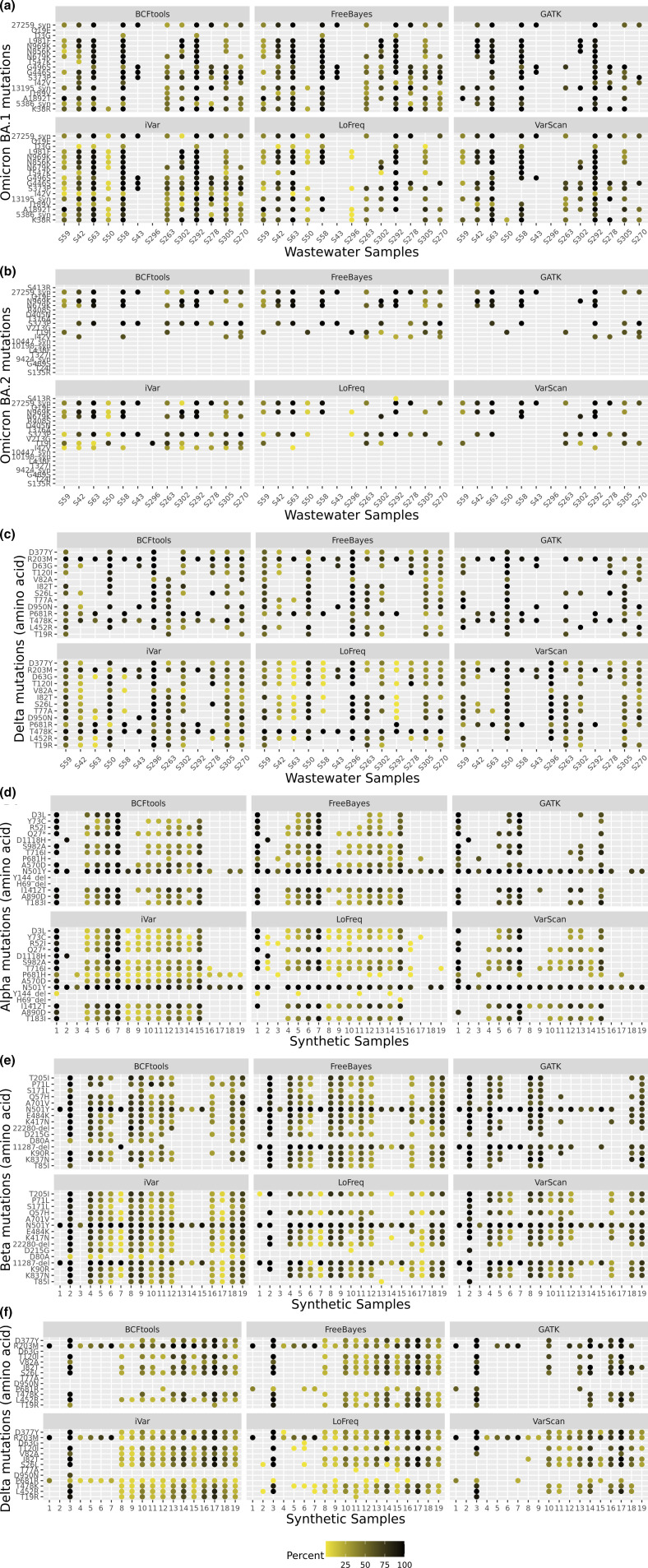
Alternate allele frequencies of the defining mutations of OmicronBA.1 (a), Omicron BA.2 (b) and Delta (c) were plotted for 13 wastewater samples for six differentvariant callers. Similarly, the alternate allele frequencies of defining mutations for the three VOCs ofinterest Alpha (d), Beta (e) and Delta (f) were plotted for the 19 selected synthetic datasets for sixdifferent variant callers. The data points are coloured based on the two-colour gradient, namelyyellow (0%) and black (100%). Degenerate codons were not plotted.

We extrapolated the alternate allele frequencies values from the vcf files for both the synthetic and wastewater samples across the six variant callers to see whether these were called similarly. Degenerate codons were not plotted for any of the callers. In Fig. 6(a–c) (wastewater) and Fig. 6(d–f) (synthetic), we plotted all the defining mutations for the VOIs across all the 19 synthetic or the 13 wastewater samples. Frequencies are coloured by gradient (Fig. 6 was also plotted in different formats as shown in Figs S8, S9 and S10).

Among the synthetic samples, all the callers had the same frequency for those samples where there was a high proportion of a variant (75–100 %), such as samples 1 and 7 for Alpha (Fig. 6d), sample 2 for Beta (Fig. 6e), and samples 3 and 17 for Delta (Fig. 6f). For the remaining samples, frequencies were similar; although iVar called more mutations than others, but at very low frequency. Similarly, for the wastewater samples we plotted frequencies for defining mutation for Omicron BA.1 (Fig. 6a), Omicron BA.2 (Fig. 6b) and Delta (Fig. 6c) variants. BA.1 mutation frequencies were called at the same level across all the callers. In particular, samples S58, S63 and S292 were the ones with the highest number of mutations detected by all callers, at the same high frequency. All three samples were found to be positive for Omicron from previous data analysis (data not shown). Some mutations, such as Q19E, were not called by any of the callers. It is worth highlighting that at the time the original samples were analysed (December 2021) there was no clear distinction between BA.1 and BA.2. Frequencies for BA.2 (Fig. 6e) were also similar across the callers, although only a subset of these were detected, suggesting that more likely the BA.1 subgroup was the one circulating at that time. As for the Delta variant, two samples, S50 and S296, showed high frequency and were consistent among all the callers. Other samples were called similarly, with no evident differences.

## Discussion

In this paper, we have analysed six variant callers commonly used in bioinformatics data analysis to empirically quantify their ability to identify mutations across mixtures of synthetic samples with known mutations as well as a set of wastewater samples with an unknown number of mutations. We first calculated recall and precision across the full genome for all the samples to define ground differences across the callers, to then focus in more detail on a set of defining mutations, by comparing how many of these were found and at which frequency by each of the callers. Our results suggest that the variant callers that showed the highest precision when looking at all samples together (synthetic and real wastewater samples) were GATK and VarScan, followed by BCFtools and FreeBayes, which instead showed sparser data points. LoFreq and iVar showed the lowest precision values (Fig. S1c). Recall values were the lowest for GATK (and very sparse values), while they were significant statistical differences among the rest of the callers (in particular iVar’s stochastic dominance). Overall, the F1 score confirmed that LoFreq was the least sensitive (Fig. S1c), presenting numbers of mutations that are magnitudes larger than the rest, and subsequently with a lower precision. However, when focusing on selected mutations, iVar identified the highest number of expected defining mutations across both synthetic and wastewater samples, compared to the other callers.

WBE has been used for many years to monitor key pathogens such as polio [41–45]. However, it has undergone a renaissance during the SARS-CoV-2 pandemic, with many tools and software specifically designed to detect the virus in wastewater and being developed in the wake of WBE monitoring [46–50]. As such, tools used to detect the virus in clinical samples were used as templates, but in many cases these did not reflect the composition of the wastewater samples accurately, e.g. the mixed strain nature as well as degradation of the viral RNA in the environment, thus, the lack of a complete genome, and more importantly the lack of a consensus sequence. These nuances impact and should inform downstream analysis at the bioinformatics level: sequences could only be a fraction of the genome, as the samples may be highly degraded, and the lack of a consensus will affect the ability to assign a variant to a sample [51, 52]. Subsequently variant analysis is limited to a shorter region in some cases and variant assignment has to happen based on specific mutations known to define a variant, as used for clinical cases [53]. For this reason, a variant caller that adapts to the type of data available is very important as it will be expected to call the mutations with higher sensitivity.

The six callers analysed in this paper have many aspects in common as well as significant differences. For example, of all the callers, LoFreq is the only one that requires base quality information to call variants, making this method much more stringent and robust than the others, but similarly prone to call many more mutations than expected [21], as seen in our results. In addition, it has been reported that LoFreq can efficiently recognize sequencing errors from expected mutations in non-environmental samples; however, it seems that it did not behave as efficiently in our wastewater samples nor with the synthetic samples. This could also be a consequence that more specific combinations of parameters have to be chosen for efficient performance. Although for some samples the recall was correct, the precision was lower indicating the lower efficiency. Indeed, LoFreq is a fast and sensitive variant caller that calls many mutations that, however, are not TP; hence, it has low recall and precision as found in our results. We suggest that this tool is more suited for shorter viral sequences, more likely in samples with higher coverage. Similarly, GATK was designed to detect genomes across a range of sample sources, but not environmental ones, and this is reflected in the highly comprehensive set of parameters available to efficiently analyse the datasets (over 111 parameters) [20], but most of GATK parameters are not applicable to wastewater datasets. These aspects also highlight the difficulty in using these two tools for wastewater data, in contrast to FreeBayes, BCFtools or VarScan, which are functional and easy to apply in non-clinical settings. Nevertheless, we found that compared to most of the callers analysed, GATK did find the majority of the expected mutations (TPs), as well as having overall good scores for recall and precision, suggesting that applying different sets of parameters might improve its functionality for environmental samples as well. This applies to LoFreq also.

The selected variant callers have been extensively used in other fields for comparison purposes. For example, a recent paper comparing the efficiency of different mappers and callers in plant NGS (next generation sequencing) data found that GATK was the best caller among those tested, suggesting that the type of data greatly affects not only the results but also the choice of tools used to analyse the datasets [30, 54, 55]. Although GATK was not the best of the callers in our study, we suggest these results are valid given the diversity of the datasets; namely, the plant genome being of better quality and not containing mixed strains compared to the wastewater; secondly, the fact that the expected mutations are not always known; thus, many more mutations will be counted as TP or FP. This is independent of the pathogen studied, since most of the tools are widely applicable in different fields. Similarly, another study looking at exome sequencing also found that among the variant callers analysed GATK UnifiedGenotyper performed best [54]. We suggest that looking at shorter regions of a genome, such as exons, has its advantage since it allows us to work with a relatively smaller and highly covered region. In addition, compared to the above study, we used GATK HaplotypeCaller, which is known to have a different algorithm for calling variants than GATK UnifiedGenotyper.

It should also be noted that many of the parameters within each of these variant callers were left in default in our analysis. In fact, the wide choice of parameters poses a risk on its own when comparing different tools as it introduces biases. In an ideal setting, the correct procedure will indicate that all parameters are tested and those reflecting an outcome that is expected are then chosen. In this work, we did not assume a certain output as we did not use all the possible parameters and because we were not expecting similar results between the callers. Indeed, a small test using FreeBayes showed us that changing certain key parameters yielded many different outputs, all of them being acceptable results (data not shown). Because these parameters are not shared or found in other callers, the comparison could not be achieved, as it would have introduced an advantage or disadvantage for some callers. This is in agreement with current literature, where on many occasions parameters are left in default [30–32]. A direct consequence of this is that many results across our data would have had a different outcome. Indeed, LoFreq results might differ enormously if we had considered and adjusted all the parameters accordingly, irrespective of whether these were common to other variant callers.

It should be noted that calculating recall and precision for samples containing a mix of variants (two or more) is a cumbersome task, as some mutations can be shared among the variants, which will affect the ground truth. At the time of writing, Deng *et al*. [35] designed a tool where only samples with a mix of two genomes can be used. However, as of now, there is no tool available for samples with a mix of three or more genomes. Wastewater is a mixed sample, sometimes containing more than two variants. However, this notion will only be confirmed overtime, through sequencing of clinical cases. Therefore, with the aim to reflect real wastewater data, we calculated recall and precision for each variant known to be prevalent as it would be at that time that specific variant would have been circulating. This will inevitably overestimate the number of FPs in each run as it should be calculated only once per sample but it is, however, expected: any position not found to be a TP for one variant will show as a FP. But, by testing each of the variants separately, this will give us the correct TPs, which are the values we have been using in our paper to compare the callers (defining mutations).

### Conclusion

In conclusion, we suggest that callers such as VarScan, BCFtools and Freebayes are overall preferable (Fig. S1c), particularly when mutations are not known, as they are called with higher specificity and sensitivity. However, if specific mutations are under investigation and expected in the output, such as the ones we used as variant-defining, iVar performed best. We also suggest that, upon choice of one variant caller for a specific study other than comparison purposes, all parameters should be explored and tested to better improve the calling capability. In the future, tools that can analyse mixed samples without the need to run the strains separately are also preferred, as they will give even more accurate values of recall and precision.

## Supplementary Data

Supplementary material 1

Supplementary material 2

Supplementary material 3

Supplementary material 4

Supplementary material 5

## References

[1] Cucinotta D, Vanelli M (2020). WHO declares COVID-19 a pandemic. Acta Biomed.

[2] Aguiar-Oliveira M de L, Campos A, R Matos A, Rigotto C, Sotero-Martins A (2020). Wastewater-based epidemiology (WBE) and viral detection in polluted surface water: a valuable tool for COVID-19 surveillance-a brief review. Int J Environ Res Public Health.

[3] Peccia J, Zulli A, Brackney DE, Grubaugh ND, Kaplan EH (2020). Measurement of SARS-CoV-2 RNA in wastewater tracks community infection dynamics. Nat Biotechnol.

[4] Sutton M, Radniecki TS, Kaya D, Alegre D, Geniza M (2022). Detection of SARS-CoV-2 B.1.351 (Beta) variant through wastewater surveillance before case detection in a community, Oregon, USA. Emerg Infect Dis.

[5] Mallapaty S (2020). How sewage could reveal true scale of coronavirus outbreak. Nature.

[6] World Health Organization (2022). Tracking SARS-CoV-2 variants. https://www.who.int/en/activities/tracking-SARS-CoV-2-variants/.

[7] NCBI (2022). SARS-CoV-2 variants overview. https://www.ncbi.nlm.nih.gov/activ.

[8] UKHSA (2010). Emerging infections: Horizon Scanning Programme. https://www.gov.uk/government/collections/emerging-infections.

[9] UKHSA (2022). Investigation of SARS-CoV-2 Variants: Technical Briefings.

[10] UKHSA (2022). UK Completes Over 2 Million SARS-CoV-2 Whole Genome Sequences.

[11] Xiao A, Wu F, Bushman M, Zhang J, Imakaev M (2022). Metrics to relate COVID-19 wastewater data to clinical testing dynamics. Water Res.

[12] Wolfe MK, Topol A, Knudson A, Simpson A, White B (2021). High-frequency, high-throughput quantification of SARS-CoV-2 RNA in wastewater settled solids at eight publicly owned treatment works in Northern California shows strong association with COVID-19 incidence. mSystems.

[13] Weidhaas J, Aanderud ZT, Roper DK, VanDerslice J, Gaddis EB (2021). Correlation of SARS-CoV-2 RNA in wastewater with COVID-19 disease burden in sewersheds. Sci Total Environ.

[14] Peinado B, Martínez-García L, Martínez F, Nozal L, Sánchez MB (2022). Improved methods for the detection and quantification of SARS-CoV-2 RNA in wastewater. Sci Rep.

[15] Ewels PA, Peltzer A, Fillinger S, Patel H, Alneberg J (2020). The nf-core framework for community-curated bioinformatics pipelines. Nat Biotechnol.

[16] Posada-Céspedes S, Seifert D, Topolsky I, Jablonski KP, Metzner KJ (2021). V-pipe: a computational pipeline for assessing viral genetic diversity from high-throughput data. Bioinformatics.

[17] Grubaugh ND, Gangavarapu K, Quick J, Matteson NL, De Jesus JG (2019). An amplicon-based sequencing framework for accurately measuring intrahost virus diversity using PrimalSeq and iVar. Genome Biol.

[18] Koboldt DC, Chen K, Wylie T, Larson DE, McLellan MD (2009). VarScan: variant detection in massively parallel sequencing of individual and pooled samples. Bioinformatics.

[19] Brown MR, Wade MJ, McIntyre-Nolan S, Bassano I, Denise H (2021). Wastewater Monitoring of SARS-CoV-2 Variants in England: Demonstration Case Study for Bristol (Dec 2020–March 2021). Summary for SAGE 08/04/21.

[20] McKenna A, Hanna M, Banks E, Sivachenko A, Cibulskis K (2010). The genome analysis toolkit: a MapReduce framework for analyzing next-generation DNA sequencing data. Genome Res.

[21] Wilm A, Aw PPK, Bertrand D, Yeo GHT, Ong SH (2012). LoFreq: a sequence-quality aware, ultra-sensitive variant caller for uncovering cell-population heterogeneity from high-throughput sequencing datasets. Nucleic Acids Res.

[22] Garrison E, Marth G (2012). Haplotype-based variant detection from short-read sequencing.

[23] Danecek P, Bonfield JK, Liddle J, Marshall J, Ohan V (2021). Twelve years of SAMtools and BCFtools. Gigascience.

[24] Olm MR, Crits-Christoph A, Bouma-Gregson K, Firek BA, Morowitz MJ (2021). inStrain profiles population microdiversity from metagenomic data and sensitively detects shared microbial strains. Nat Biotechnol.

[25] Costea PI, Munch R, Coelho LP, Paoli L, Sunagawa S (2017). metaSNV: a tool for metagenomic strain level analysis. PLoS One.

[26] Jeffries A, Child HT, Paterson S, Loose M, van Aerle R (2022). Wastewater sequencing using the EasySeq RC-PCR SARS CoV-2 (Nimagen) V2.0 V.2. 2022. https://www.protocols.io/view/wastewater-sequencing-using-the-easyseq-rc-pcr-sar-81wgb7bx3vpk/v2.

[27] Loman N, Rowe W, Rambau A (2020). nCoV-2019 novel coronavirus bioinformatics protocol. https://artic.network/ncov-2019/ncov2019-bioinformatics-sop.html.

[28] Krueger F, James F, Ewels P, Afyounian E, Schuster-Boeckler B (2021). Trim Galore. https://zenodo.org/record/5127899#.YoQSyXXMI2w.

[29] Li H, Durbin R (2009). Fast and accurate short read alignment with Burrows-Wheeler transform. Bioinformatics.

[30] Schilbert HM, Rempel A, Pucker B (2020). Comparison of read mapping and variant calling tools for the analysis of plant NGS data. Plants.

[31] Xu C (2018). A review of somatic single nucleotide variant calling algorithms for next-generation sequencing data. Comput Struct Biotechnol J.

[32] Sandmann S, de Graaf AO, Karimi M, van der Reijden BA, Hellström-Lindberg E (2017). Evaluating variant calling tools for non-matched next-generation sequencing data. Sci Rep.

[33] Albers CA, Lunter G, MacArthur DG, McVean G, Ouwehand WH (2011). Dindel: accurate indel calls from short-read data. Genome Res.

[34] Patel H, Varona S, Monzón S, Espinosa-Carrasco J, Heuer ML (2022). nf-core/viralrecon: nf-core/viralrecon v2.5 – Manganese Monkey. https://zenodo.org/record/6827984#.Yxm4OKHMI2w.

[35] Deng Z-L, Dhingra A, Fritz A, Götting J, Münch PC (2021). Evaluating assembly and variant calling software for strain-resolved analysis of large DNA viruses. Brief Bioinform.

[36] Schmidt J, Berghaus S, Blessing F, Herbeck H, Blessing J (2022). Genotyping of familial Mediterranean fever gene (*MEFV*)–single nucleotide polymorphism–comparison of Nanopore with conventional Sanger sequencing. PLoS One.

[37] Parikh R, Mathai A, Parikh S, Chandra Sekhar G, Thomas R (2008). Understanding and using sensitivity, specificity and predictive values. Indian J Ophthalmol.

[38] Olson ND, Lund SP, Colman RE, Foster JT, Sahl JW (2015). Best practices for evaluating single nucleotide variant calling methods for microbial genomics. Front Genet.

[39] Wickham H (2016). ggplot2: Elegant Graphics for Data Analysis.

[40] Garrison E, Kronenberg ZN, Dawson ET, Pedersen BS, Prins P (2021). Vcflib and tools for processing the VCF variant call format. bioRxiv.

[41] Pogka V, Labropoulou S, Emmanouil M, Voulgari-Kokota A, Vernardaki A (2017). Laboratory surveillance of polio and other enteroviruses in high-risk populations and environmental samples. Appl Environ Microbiol.

[42] Pavlov DN, Van Zyl WB, Van Heerden J, Grabow WOK, Ehlers MM (2005). Prevalence of vaccine-derived polioviruses in sewage and river water in South Africa. Water Res.

[43] Paul JR, Trask JD, Gard S (1940). Poliomyelitic virus in urban sewage. J Exp Med.

[44] Nakamura T, Hamasaki M, Yoshitomi H, Ishibashi T, Yoshiyama C (2015). Environmental surveillance of poliovirus in sewage water around the introduction period for inactivated polio vaccine in Japan. Appl Environ Microbiol.

[45] Metcalf TG, Melnick JL, Estes MK (1995). Environmental virology: from detection of virus in sewage and water by isolation to identification by molecular biology – a trip of over 50 years. Annu Rev Microbiol.

[46] Tran HN, Le GT, Nguyen DT, Juang R-S, Rinklebe J (2021). SARS-CoV-2 coronavirus in water and wastewater: a critical review about presence and concern. Environ Res.

[47] La Rosa G, Bonadonna L, Lucentini L, Kenmoe S, Suffredini E (2020). Coronavirus in water environments: occurrence, persistence and concentration methods – a scoping review. Water Res.

[48] Kitajima M, Ahmed W, Bibby K, Carducci A, Gerba CP (2020). SARS-CoV-2 in wastewater: state of the knowledge and research needs. Sci Total Environ.

[49] Foladori P, Cutrupi F, Segata N, Manara S, Pinto F (2020). SARS-CoV-2 from faeces to wastewater treatment: what do we know? A review. Sci Total Environ.

[50] Ahmed W, Angel N, Edson J, Bibby K, Bivins A (2020). First confirmed detection of SARS-CoV-2 in untreated wastewater in Australia: a proof of concept for the wastewater surveillance of COVID-19 in the community. Sci Total Environ.

[51] Sangkham S (2021). A review on detection of SARS-CoV-2 RNA in wastewater in light of the current knowledge of treatment process for removal of viral fragments. J Environ Manage.

[52] Corpuz MVA, Buonerba A, Vigliotta G, Zarra T, Ballesteros F (2020). Viruses in wastewater: occurrence, abundance and detection methods. Sci Total Environ.

[53] Jahn K, Dreifuss D, Topolsky I, Kull A, Ganesanandamoorthy P (2022). Early detection and surveillance of SARS-CoV-2 genomic variants in wastewater using COJAC. Nat Microbiol.

[54] Cornish A, Guda C (2015). A comparison of variant calling pipelines using genome in a bottle as a reference. Biomed Res Int.

[55] Bian X, Zhu B, Wang M, Hu Y, Chen Q (2018). Comparing the performance of selected variant callers using synthetic data and genome segmentation. BMC Bioinformatics.

