# Supplementary material for: Evaluation of variant calling algorithms for wastewater-based epidemiology using mixed populations of SARS-CoV-2 variants in synthetic and wastewater samples
Source: Microb Genom. 2023 Apr 19;9(4):mgen000933. doi: 10.1099/mgen.0.000933 (PMC10210938; doi:10.1099/mgen.0.000933)
Supplement: Supplementary material 1 [file mgen-9-933-s001.pdf]

Supplementary Figure 1A-C Statistical analysis of recall, precision and F1 score across the six variant callers for synthetic and wastewater samples. The figure shows boxplots showing variant caller scores for wastewater samples. For each figure, Top: Precision. Middle: Recall. Bottom: F1 Score. A. Boxplots showing variant caller scores for synthetic samples. Right: Post-hoc Dunn's test p-values, highlighted where $p < 0.05$ indicating significant difference between the distribution of scores of that caller with another. LoFreq is stochastically dominated by the others when evaluating precision and F1 scores. iVar is stochastically dominant for recall. B. Left: Boxplots showing variant caller scores for wastewater samples. Right: Post-hoc Dunn's test p-values, highlighted where $p < 0.05$ indicating significant difference between the distribution of scores of that caller with another. LoFreq is stochastically dominated by the others when evaluating precision and F1 scores. iVar is stochastically dominant for recall but is dominated by all callers except LoFreq for precision. C. Left: Boxplots showing variant caller scores for synthetic and wastewater samples combined. Right: Post-hoc Dunn's test p-values, highlighted where $p < 0.05$ indicating significant difference between the distribution of scores of that caller with another. LoFreq is stochastically dominated by the others when evaluating precision and F1 scores. iVar is stochastically dominant for recall but is dominated by all callers except LoFreq for precision.

Figure S1 is provided in a separate file.

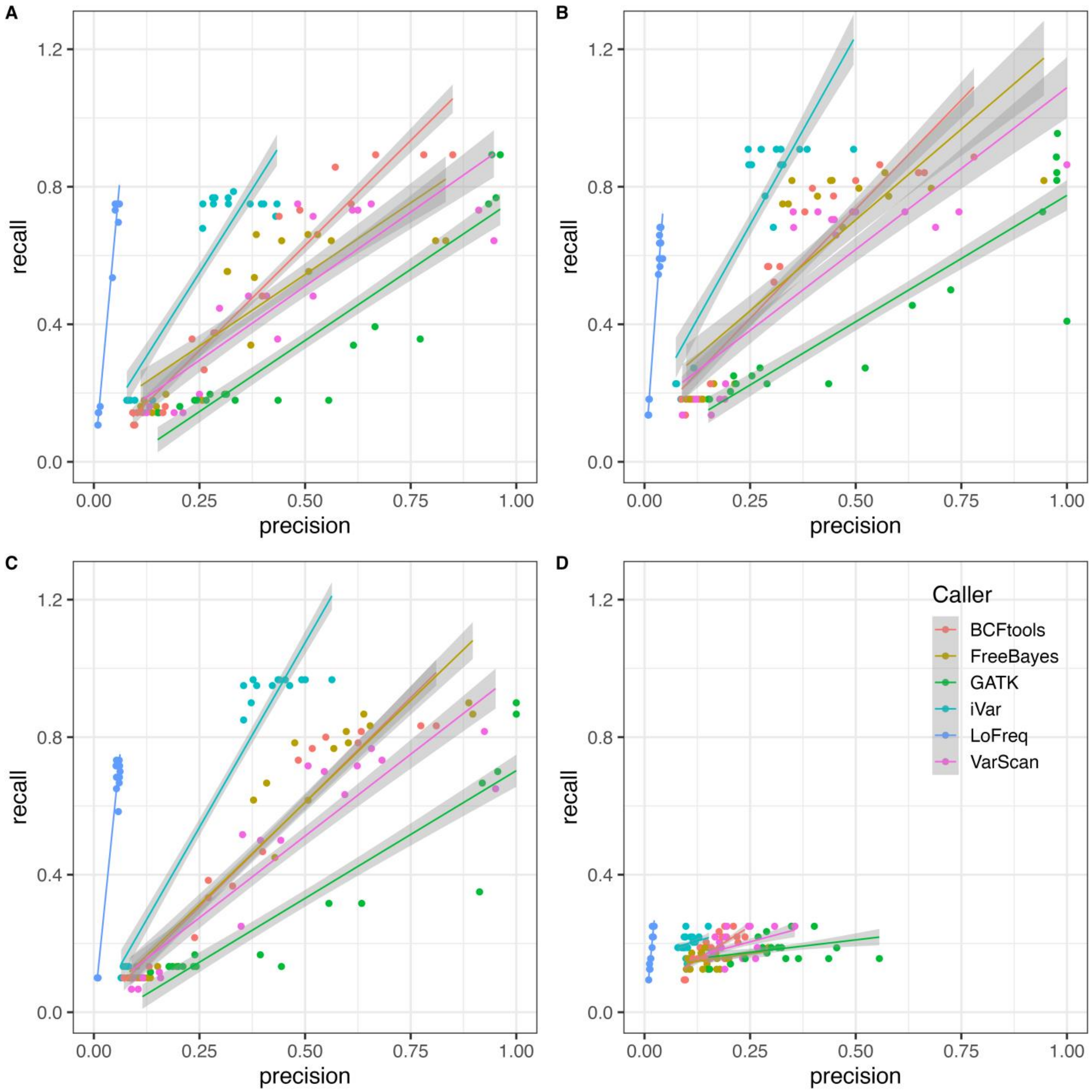

Supplementary Figure 2A-D Point plots of precision vs recall for synthetic samples, grouped and coloured by variant caller and a linear regression for each. A, Alpha VOC reference, B. Beta VOC reference, C. Delta VOC reference and D. Gamma VOC reference. The figure shows the low precision recorded by LoFreq compared to the rest of the variant callers while is higher for VarScan, FreeBayes, BCFtools and GATK. Interestingly, trend lines overlap for Freebayes and BCFtools.

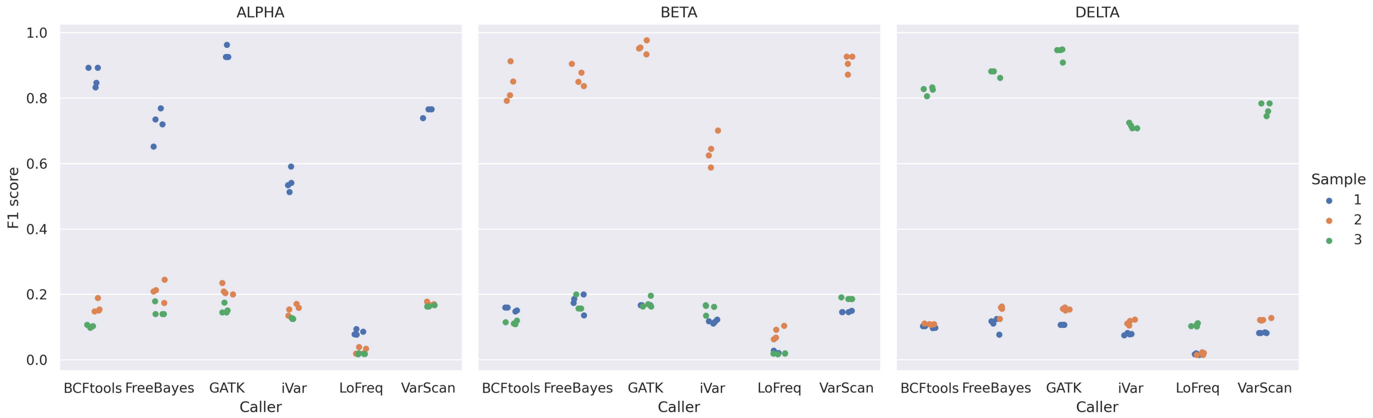

Supplementary Figure 3 Plot of F1 score vs callers for three synthetic samples (1) 100% alpha (2) 100% Beta and (3) 100% Delta as listed in Table 2 (samples with *). Each plot represents caller in relation to either the precision or the recall, expressed as the F1 score. The figure shows that all the replicates (filled circle) have similar recall and precision, suggesting that no major differences are observed. As shown in Figure 1 and 2, LoFreq shows the lowest F1 score compared to the rest of the callers, followed by iVar. FreeBayes and VarScan, showed similar results, while GATK had the highest F1 score, followed by BCFtools.

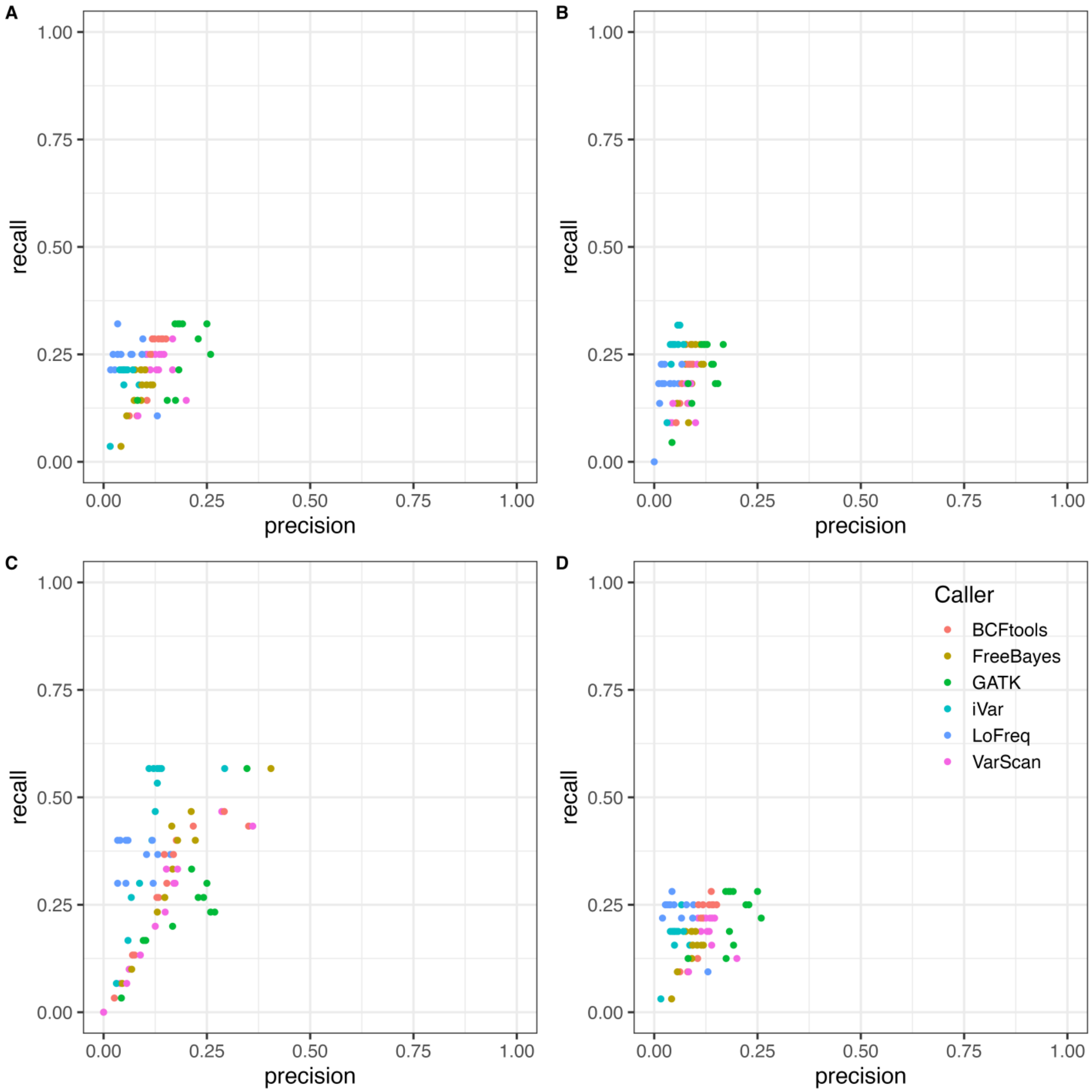

Supplementary Figure 4A-D Point plots of precision vs recall for wastewater samples, coloured by variant caller. A, Alpha VOC reference, B. Beta VOC reference, C. Delta VOC reference and D. Gamma VOC reference. The figure shows that VOCs not found in the mix such as Alpha, Beta and Gamma have low precision and recall. Some of the samples are known to be positive for the Delta VOC therefore the latter will have a higher precision and/or recall.

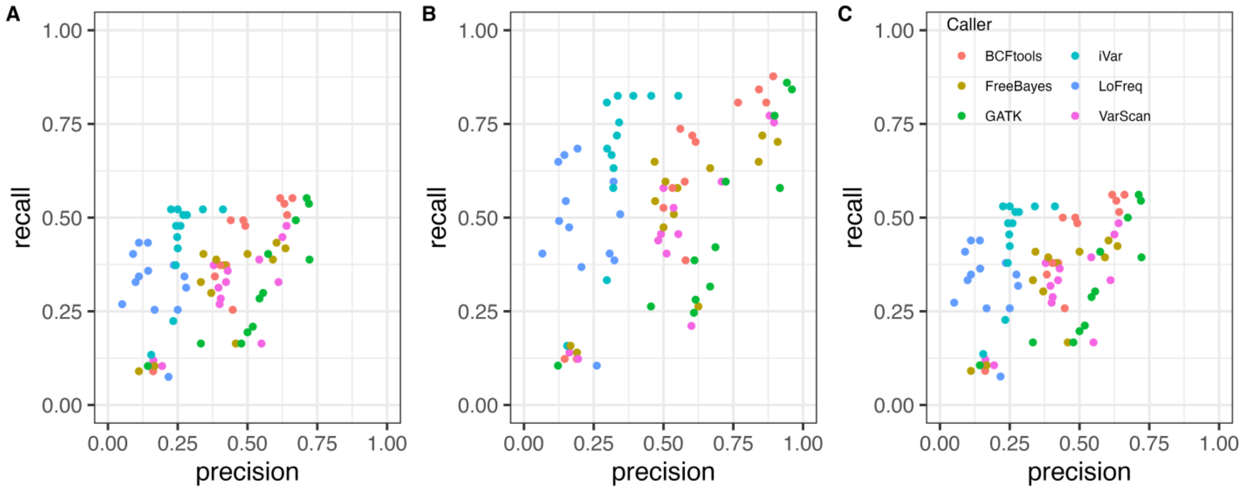

Supplementary Figure 5A-C Point plots of precision vs recall for wastewater samples for the Omicron VOC, grouped and coloured by variant caller and a linear regression for each. A, Omicron England VOC reference, B. Omicron Hong Kong VOC reference, C. Omicron Australia VOC reference. Since the wastewater samples are known to contain the Omicron VOC, samples do show a higher precision and recall compared to the negative controls used to generate Figure 4 A-B-D.

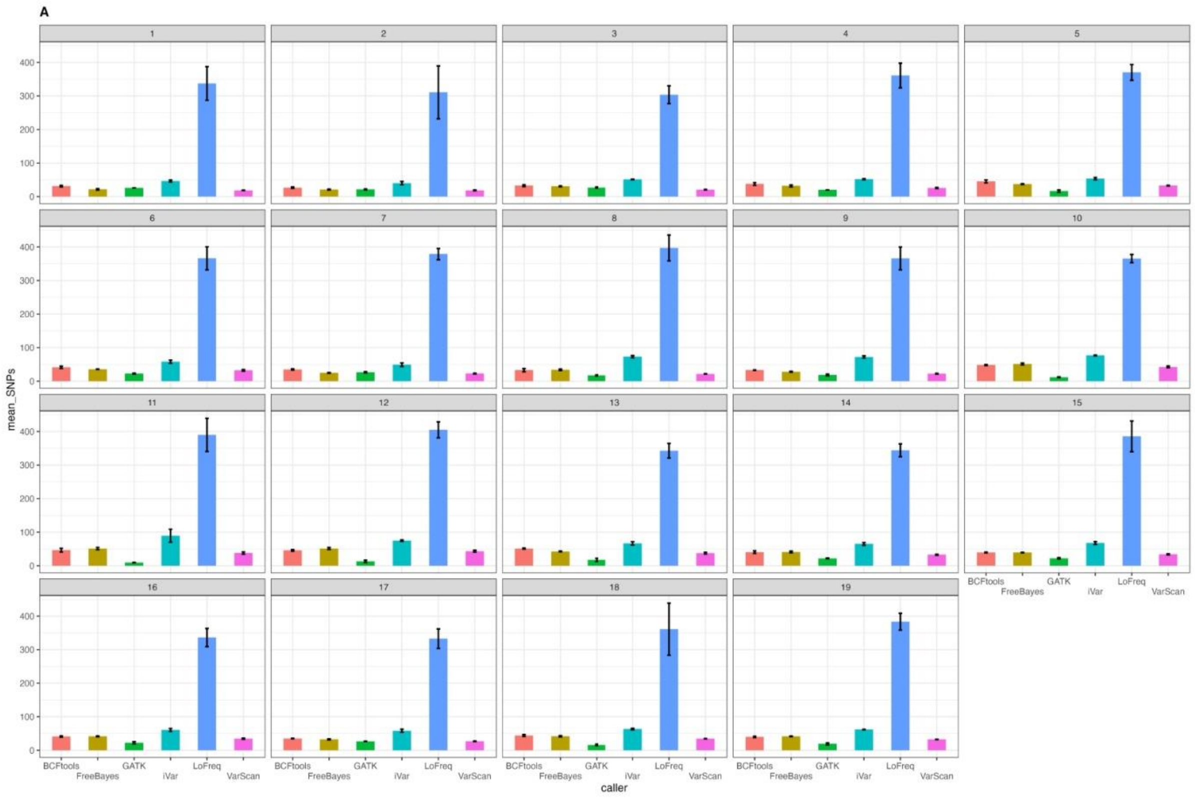

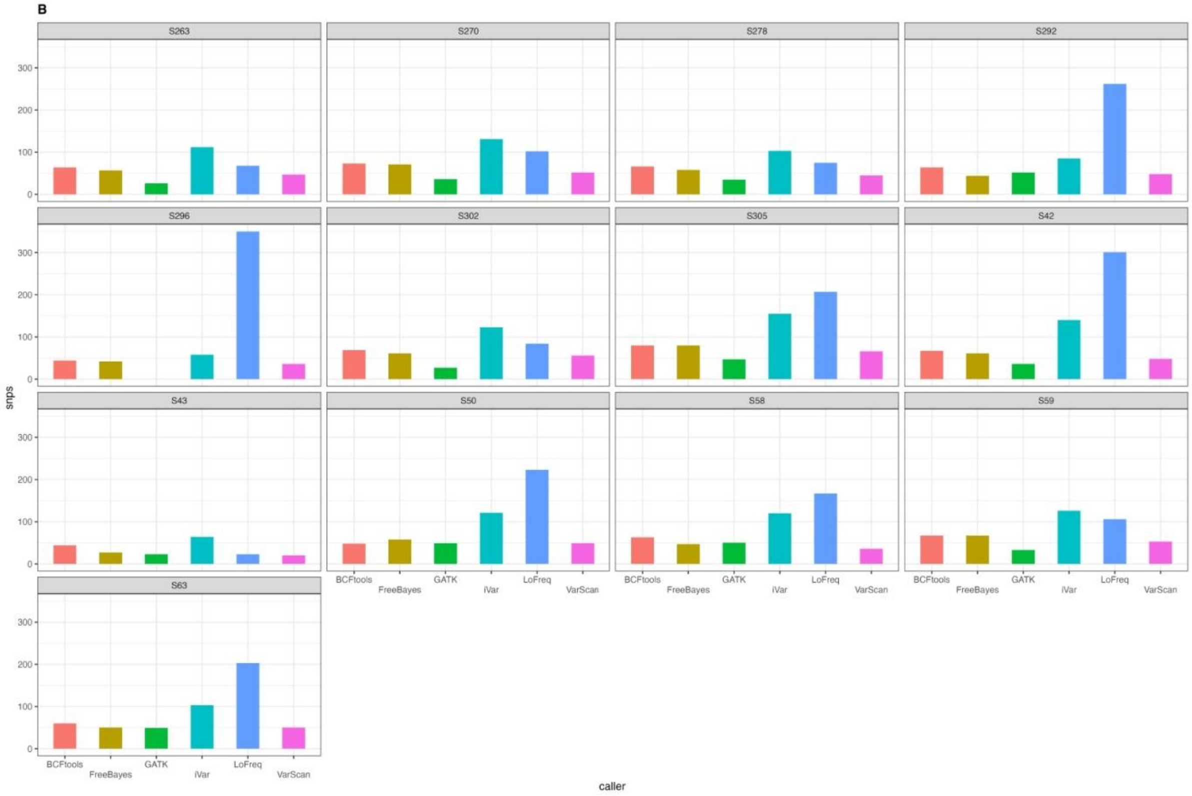
B
S263
S270
S278
S292
S296
S302
S305
S42
S43
S50
S58
S59
S63
snps
0
100
200
300
BCFtools
FreeBayes
GATK
iVar
LoFreq
VarScan
caller

c

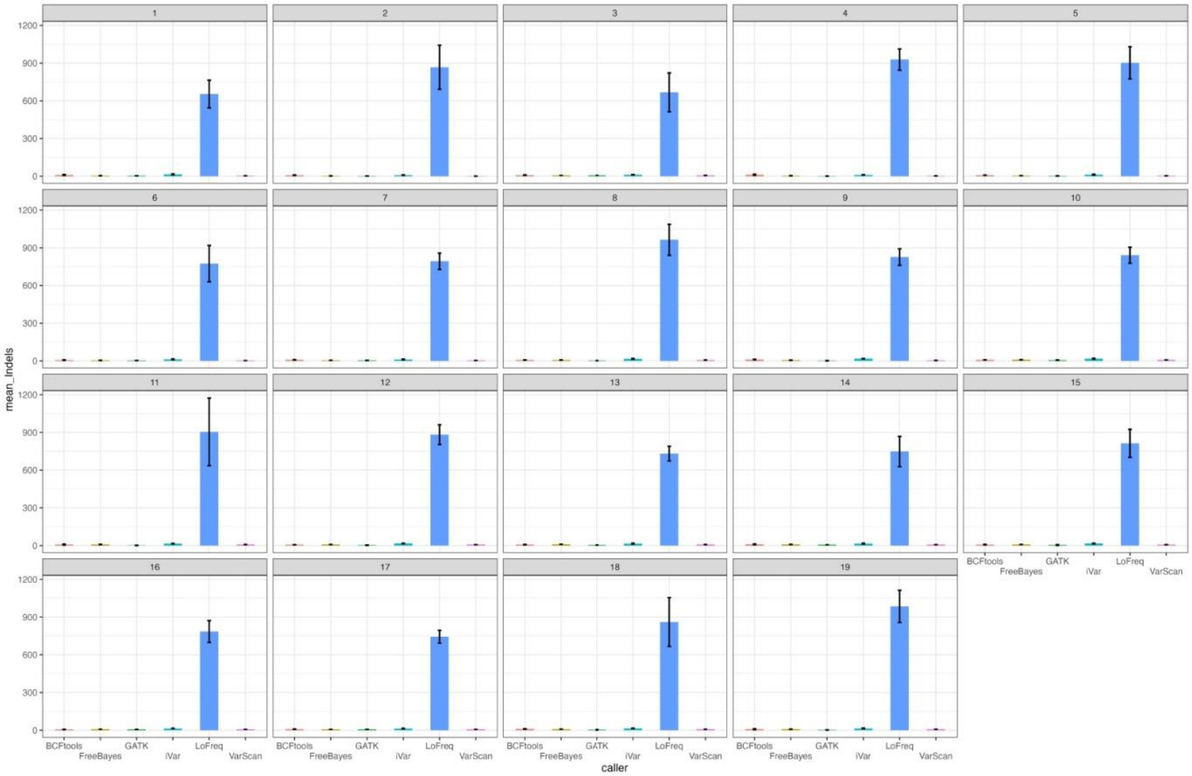
1
2
3
4
5
6
7
8
9
10
11
12
13
14
15
16
17
18
19
mean_Indels
0
300
600
900
1200
BCFtools
FreeBayes
GATK
iVar
LoFreq
VarScan
caller

D

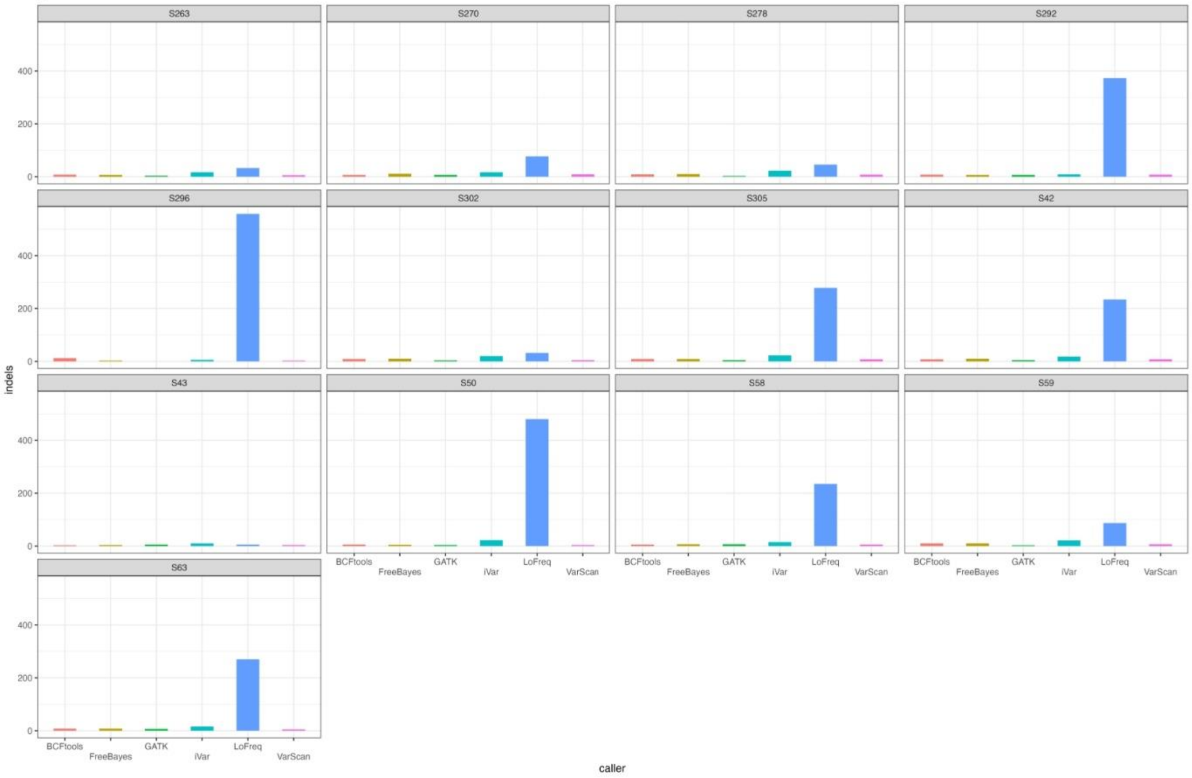
S263
S270
S278
S292
S296
S302
S305
S42
S43
S50
S58
S59
S63
indels
0
200
400
BCFtools
FreeBayes
GATK
iVar
LoFreq
VarScan
caller

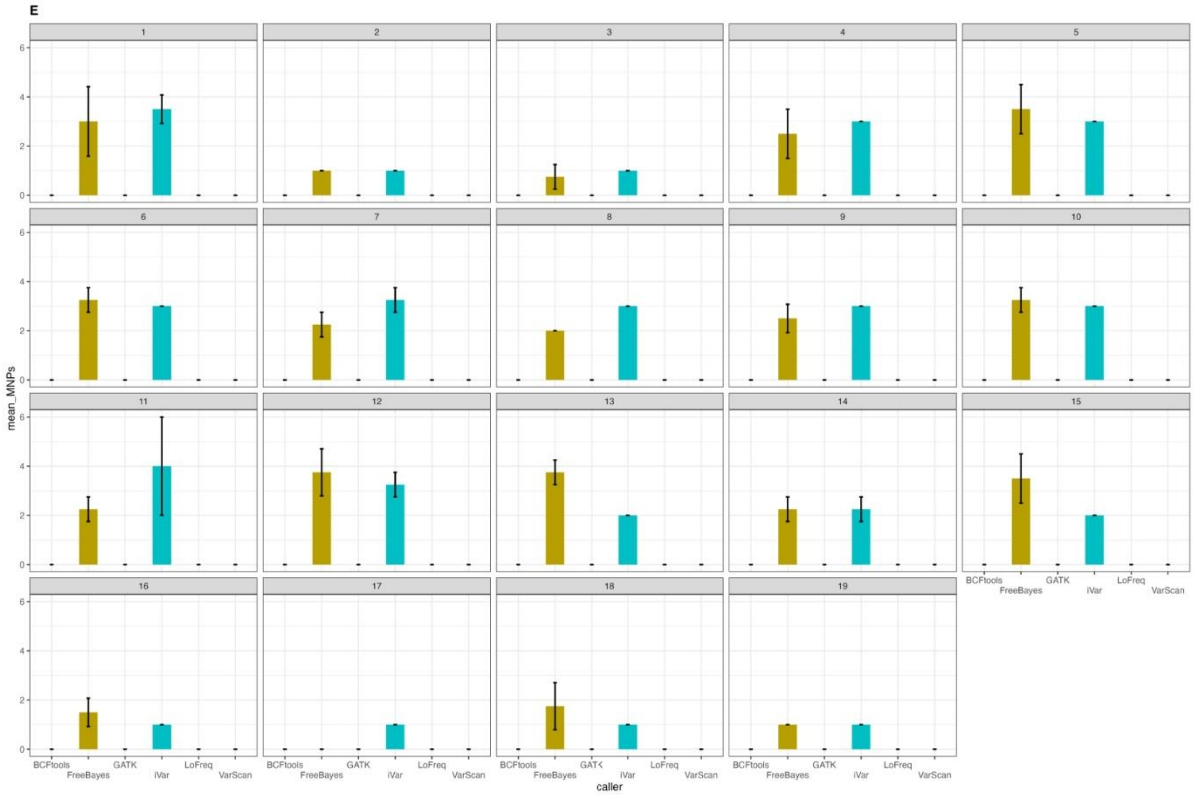
E
1
2
3
4
5
6
7
8
9
10
11
12
13
14
15
16
17
18
19
mean_MNPs
0
2
4
6
BCFtools
FreeBayes
GATK
iVar
LoFreq
VarScan
caller

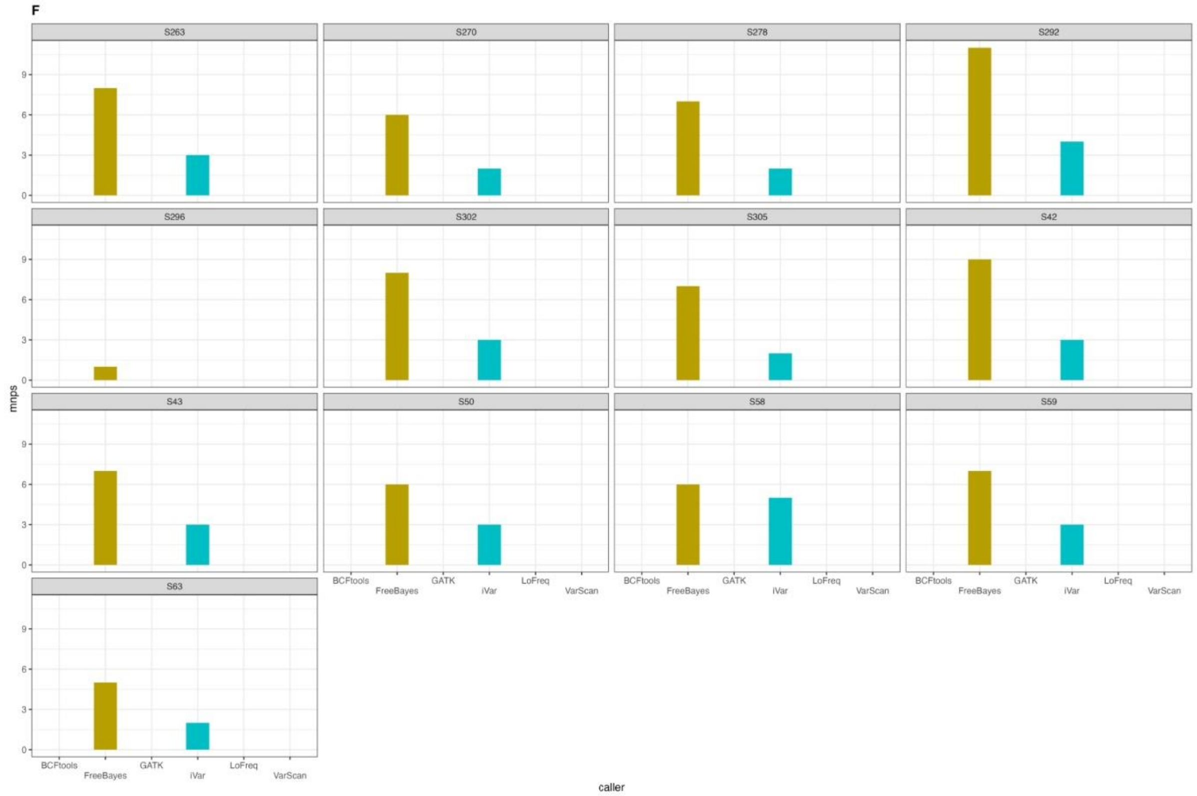

Supplementary Figure 6A-F SNPs, Indels, MNVs bar plots for synthetic and real wastewater samples.

A. Number of SNPs calculated for each of the 19 synthetic samples (mean of replicates). The figure clearly identifies LoFreq as the caller with the highest number of SNPs detected, while the rest of the callers do show a similar pattern. Detailed differences excluding LoFreq can be appreciated in Supplementary Figure 3A-B.

B. Number of SNPs calculated for each of the 13 wastewater samples with variable results among the callers. In comparison to other callers, LoFreq still calls more SNPs than expected in some of the samples, namely S296, S292, S42, S50.

C. Number of Indels calculated for each of the 19 synthetic samples (mean of replicates). As seen for the SNPs bar plots, LoFreq calls the highest number of Indels detected, while the rest of the callers do show a similar pattern. Detailed differences excluding LoFreq can be appreciated in Supplementary Figure 3C-D.

D. Number of Indels calculated for each of the 13 wastewater samples with variable results among the callers. In comparison to other callers, LoFreq still calls more Indels than expected in some of the samples, namely S296, S292, S305, S42, S50, S58 and S63. Notably, we were not able to verify the presence of Indels for GATK for sample S296.

E. Number of MNPs calculated for each of the 19 synthetic samples (mean of replicates). Only Freebayes and iVAR had detectable values to be plotted, while the rest of the callers did not call this type of base variation.

F. Number of MNPs calculated for each of the 13 wastewater samples. As seen for the synthetic samples, only Freebayes and iVAR detected the presence of MNPs.

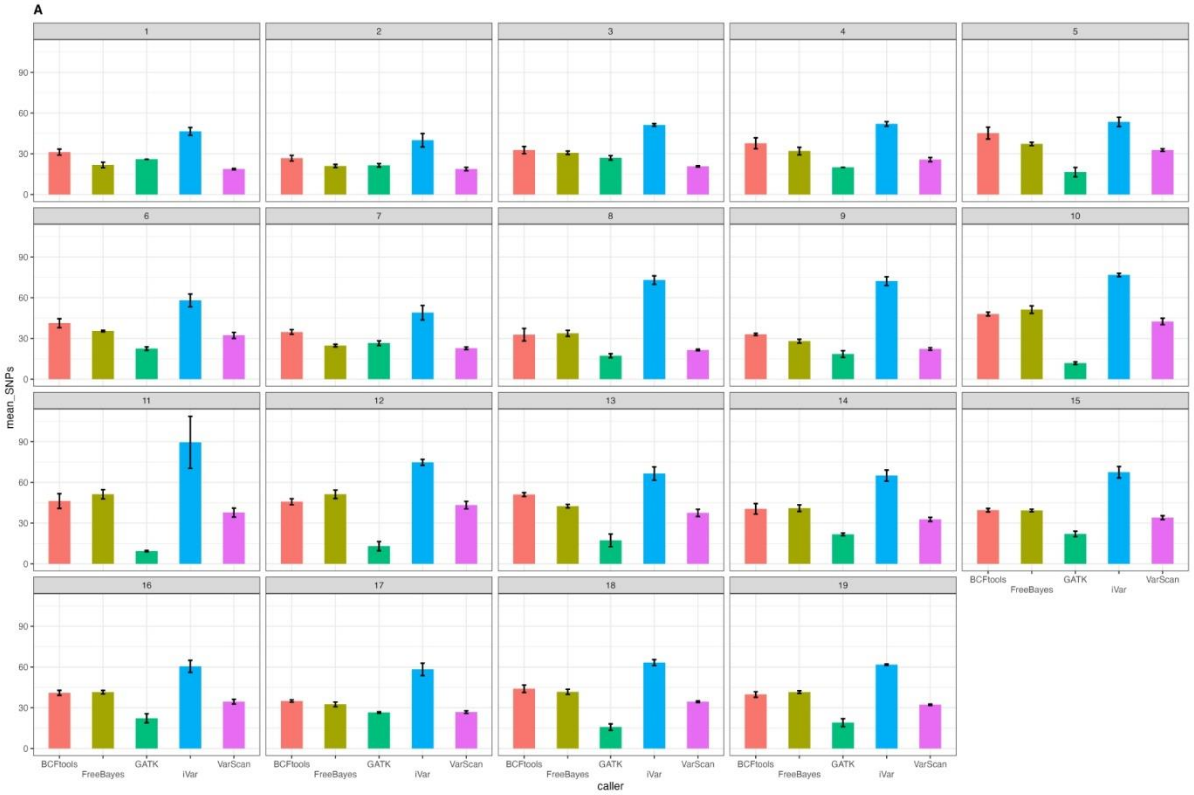

B

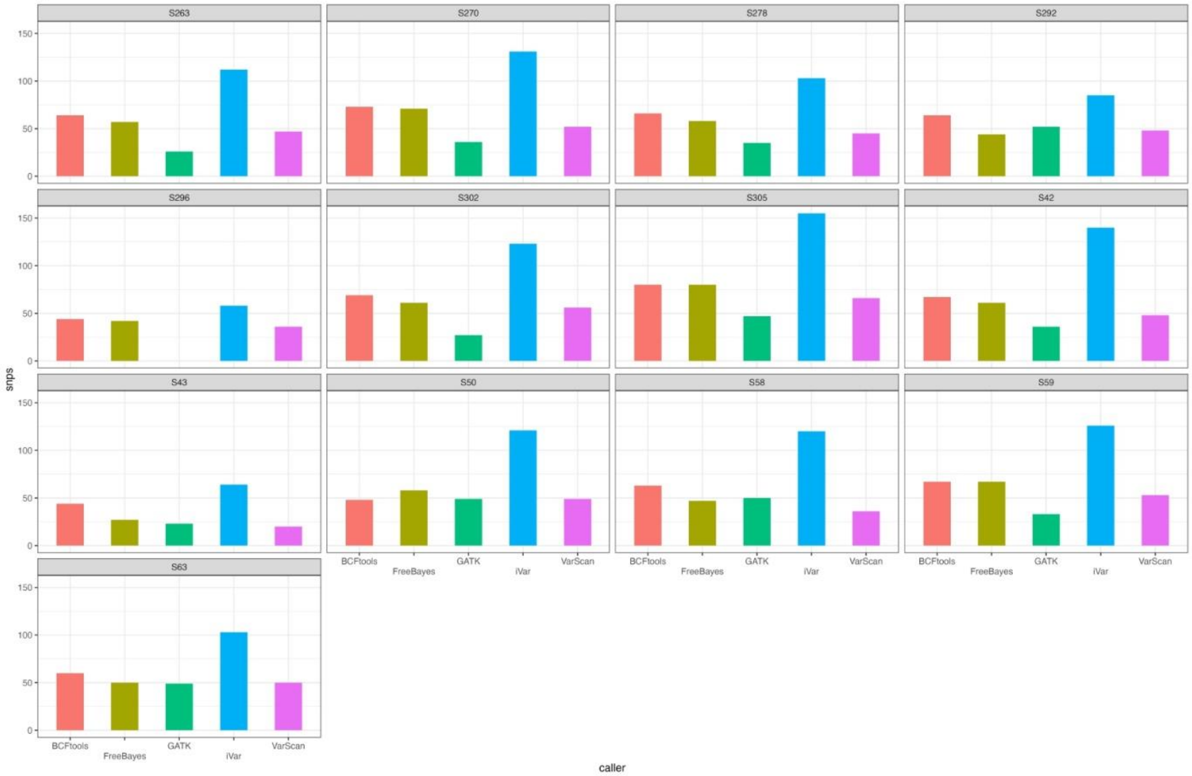
S263
S270
S278
S292
S296
S302
S305
S42
S43
S50
S58
S59
S63
snps
0
50
100
150
BCFtools
FreeBayes
GATK
iVar
VarScan
caller

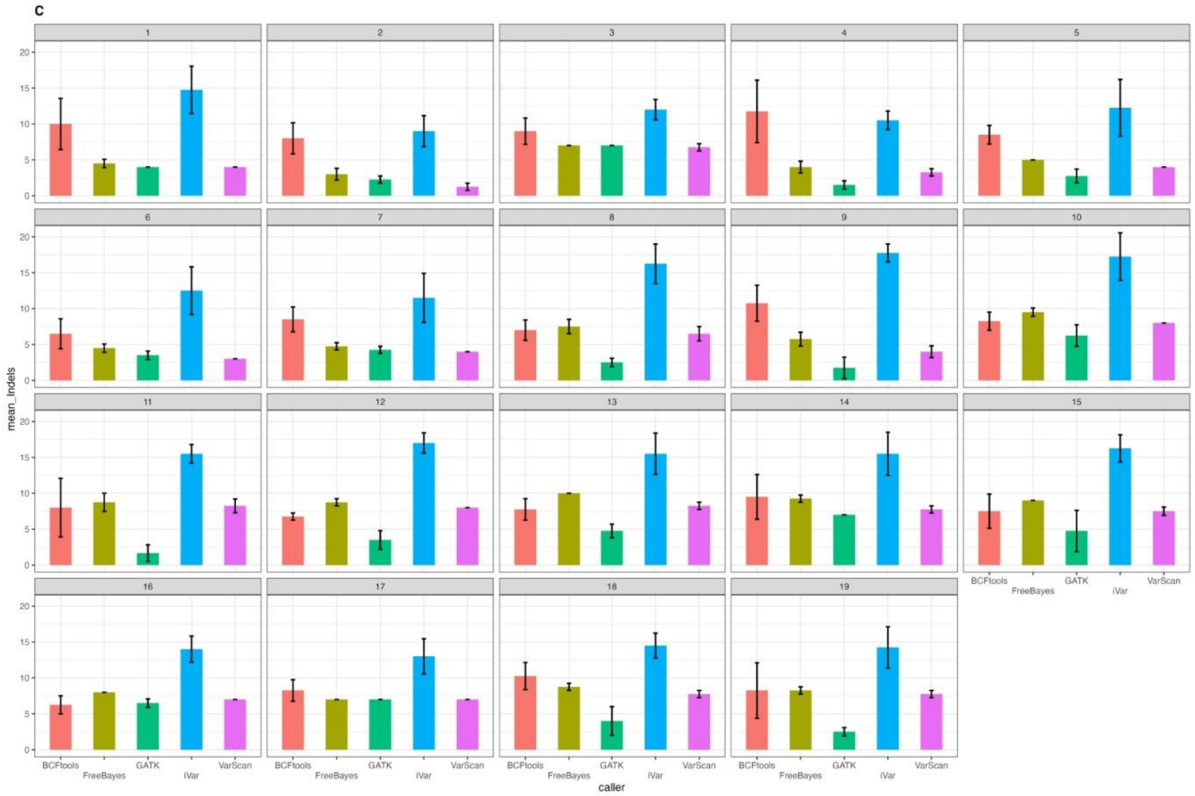
c
mean_Indels
caller
BCFtools
FreeBayes
GATK
iVar
VarScan

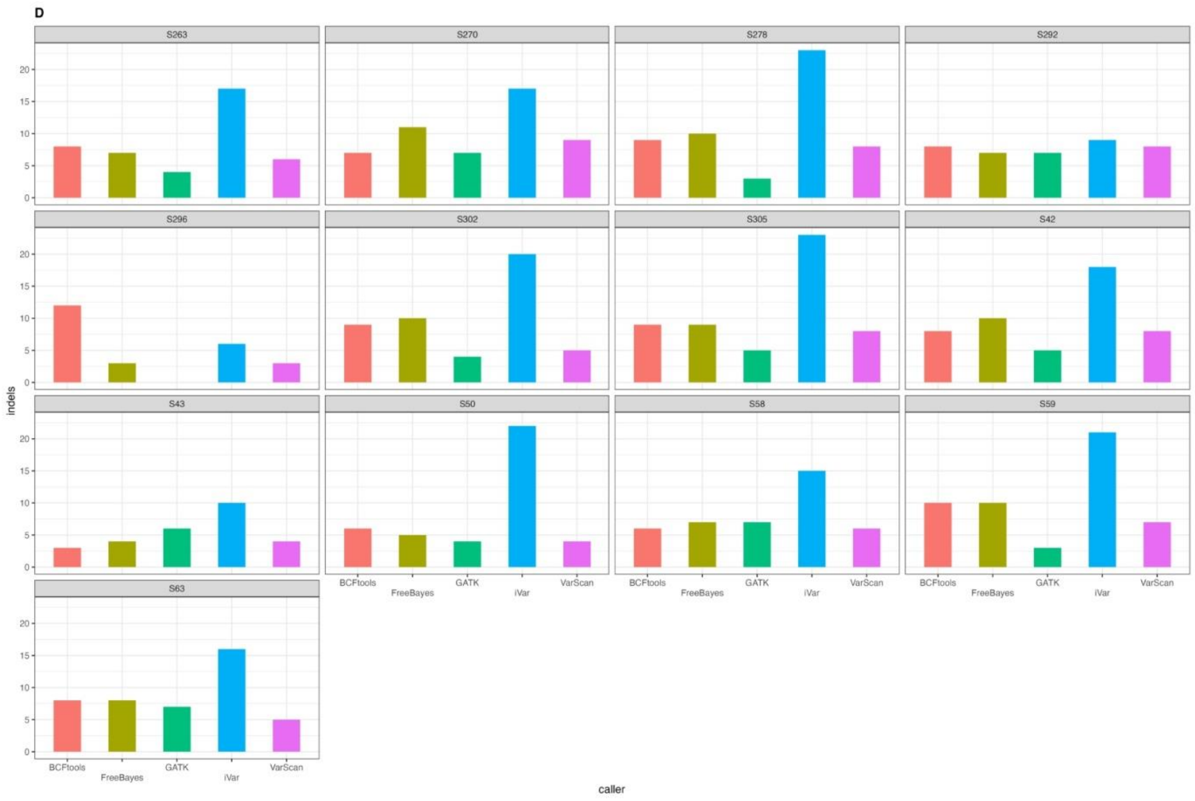
D
S263
S270
S278
S292
S296
S302
S305
S42
S43
S50
S58
S59
S63
indels
caller
BCFtools
FreeBayes
GATK
iVar
VarScan
0
5
10
15
20

E

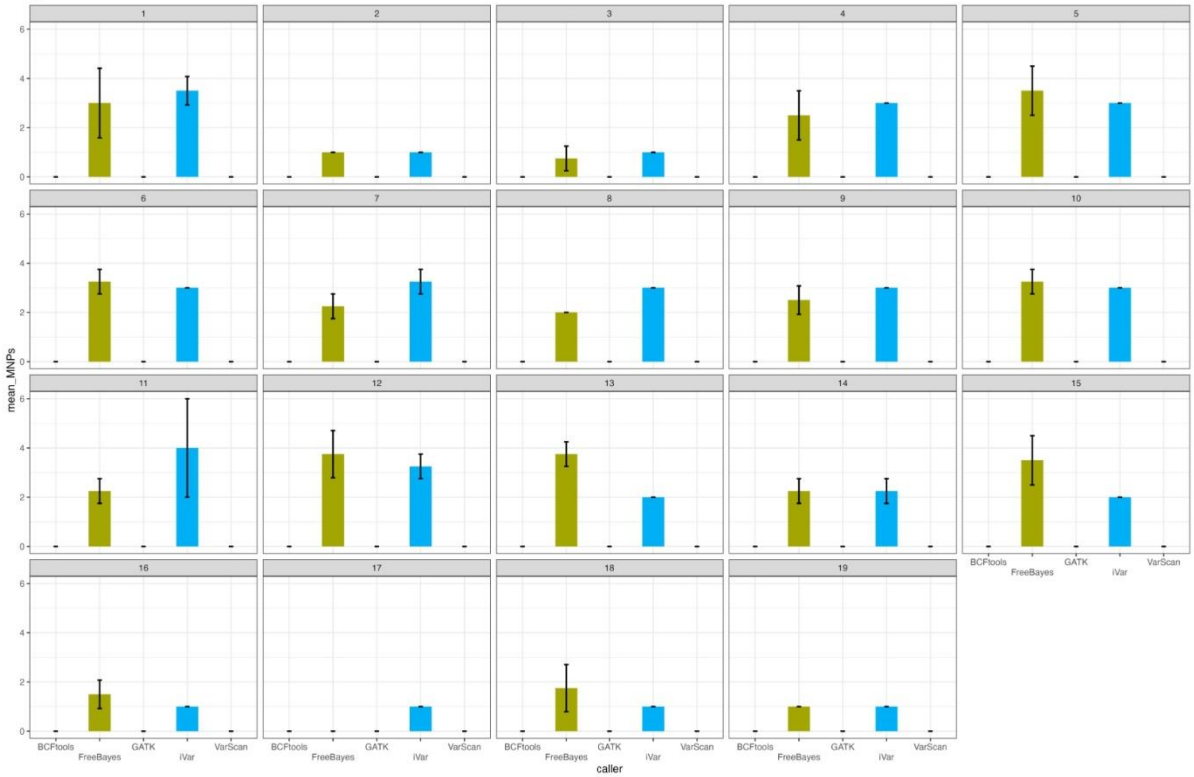

1
2
3
4
5
6
7
8
9
10
11
12
13
14
15
16
17
18
19
mean_MNPs
0
2
4
6
BCFtools
FreeBayes
GATK
iVar
VarScan
caller

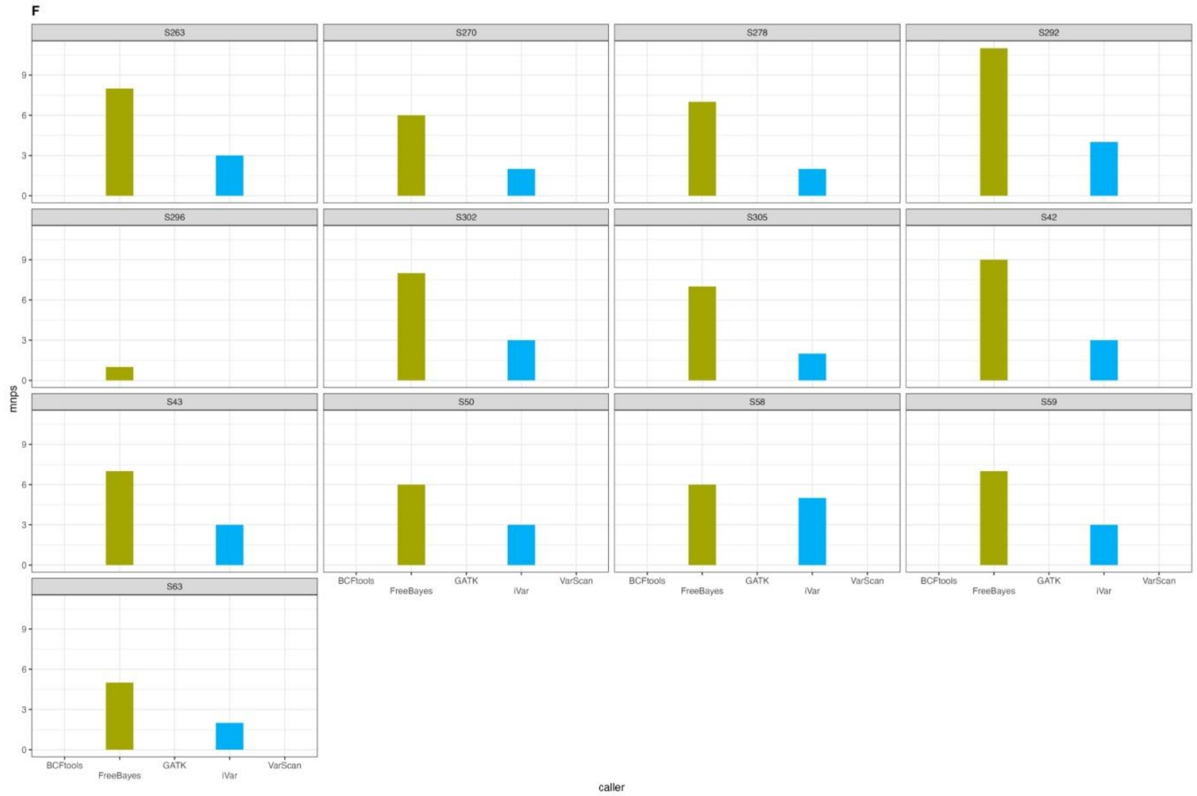

Supplementary Figure 7A-F SNPs, Indels, MNVs bar plots for synthetic and real wastewater samples without plotting LoFreq values. A. Number of SNPs calculated for each of the 19 synthetic samples (mean of replicates). B. Number of SNPs calculated for each of the 13 wastewater samples. C. Number of Indels calculated for each of the 19 synthetic samples (mean of replicates). D. Number of Indels calculated for each of the 13 wastewater samples. E. Number of MNPs calculated for each of the 19 synthetic samples (mean of replicates). F. Number of MNPs calculated for each of the 13 wastewater samples.

Fig. S8

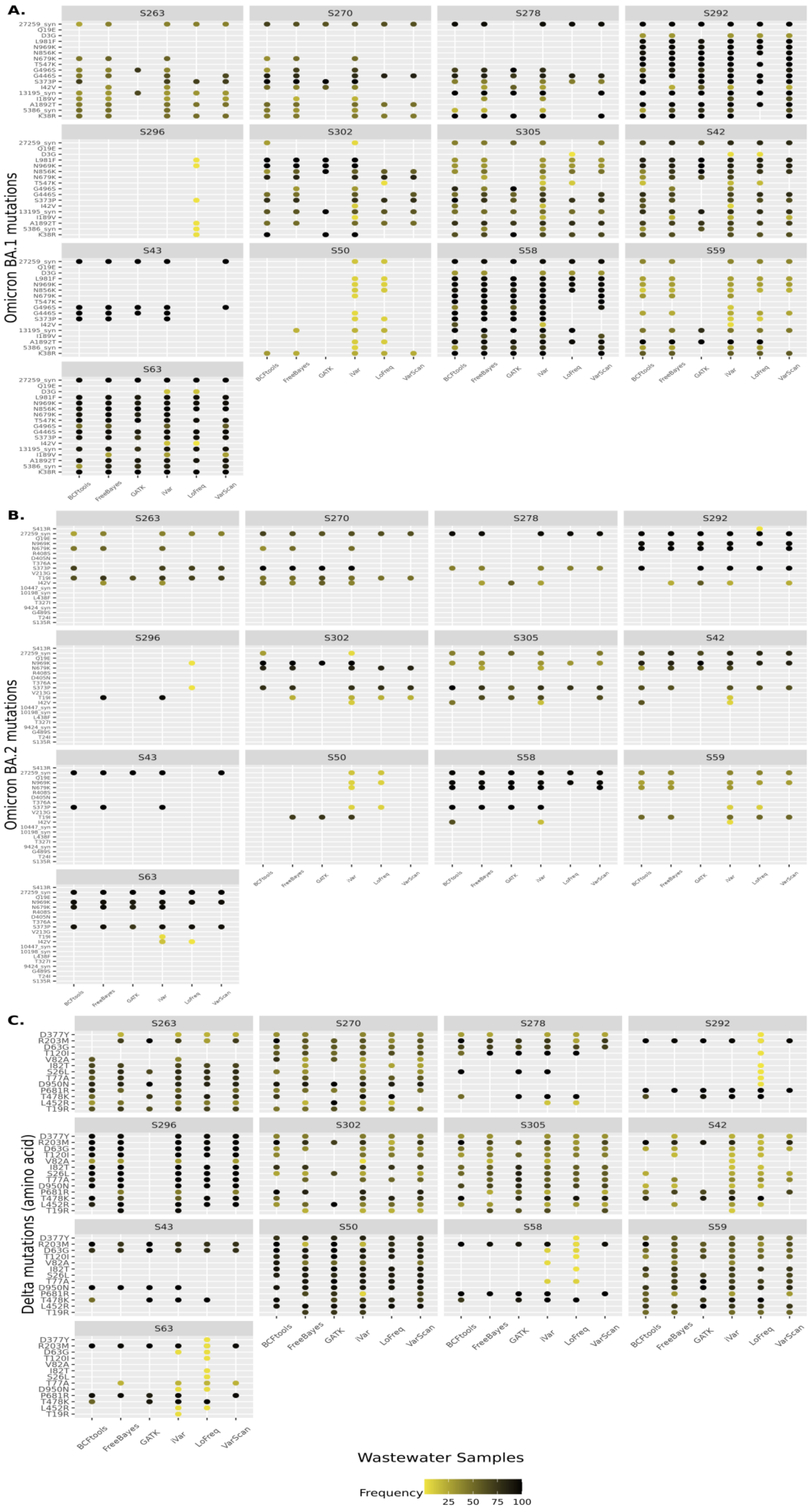
A.
B.
C.
S263
S270
S278
S292
S296
S302
S305
S42
S43
S50
S58
S59
S63
Omicron BA.1 mutations
27259_syn
Q19E
D3G
L981F
N969K
N856K
N679K
T547K
G496S
G446S
S373P
I42V
13195_syn
I189V
A1892T
5386_syn
K38R
Omicron BA.2 mutations
S413R
27259_syn
Q19E
N969K
N679K
R408S
D405N
T376A
S373P
V213G
T19I
I42V
10447_syn
10198_syn
L438F
T327I
9424_syn
G489S
T24I
S135R
Delta mutations (amino acid)
D377Y
R203M
D63G
T120I
V82A
I82T
S26L
T77A
D950N
P681R
T478K
L452R
T19R
BCFtools
FreeBayes
GATK
iVar
LoFreq
VarScan
Wastewater Samples
Frequency
25
50
75
100

Fig. S9

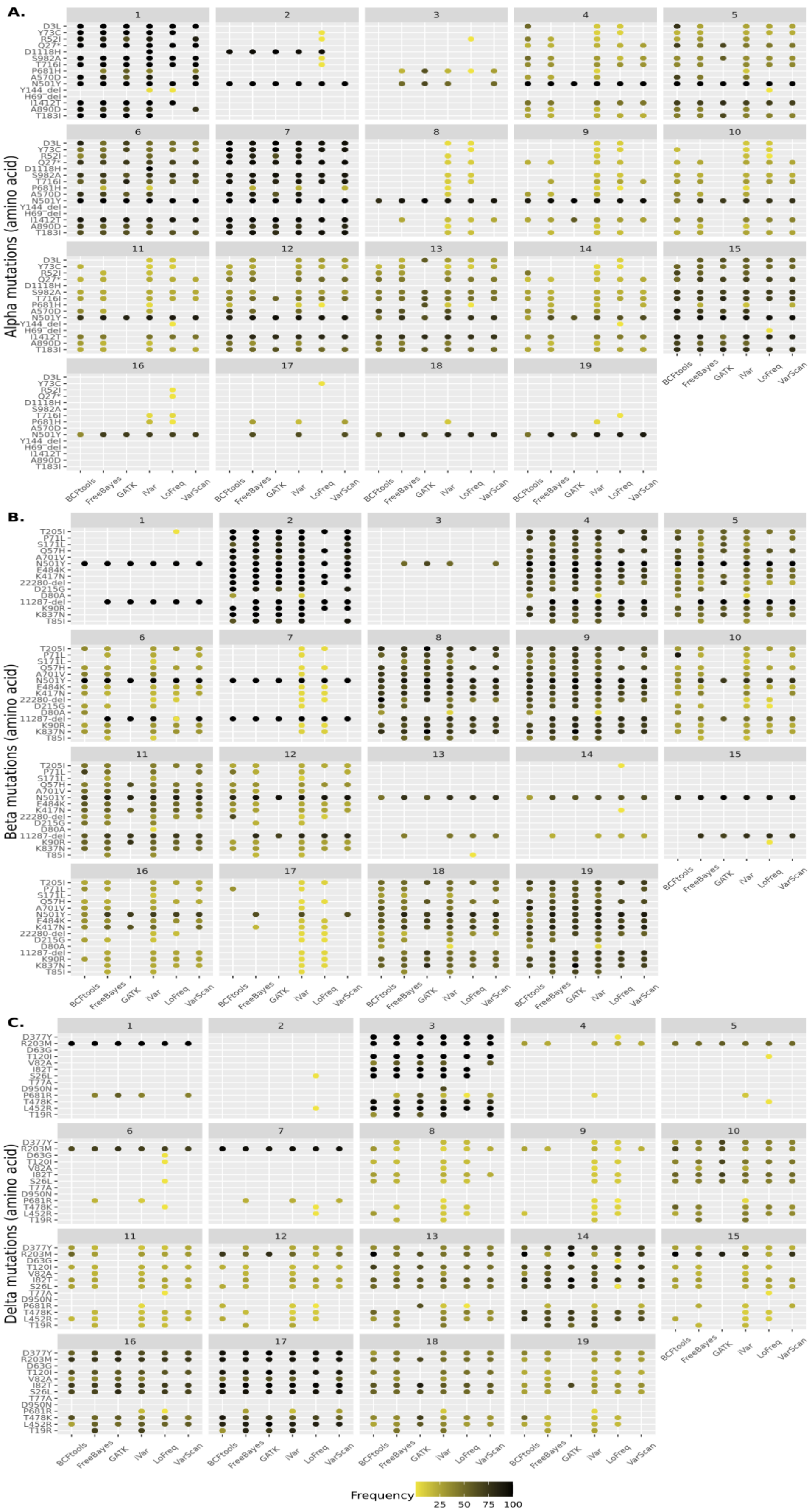
A.
Alpha mutations (amino acid)
D3L
Y73C
R52I
Q27*
D1118H
S982A
T716I
P681H
A570D
N501Y
Y144_del
H69_del
I1412T
A890D
T183I
B.
Beta mutations (amino acid)
T205I
P71L
S171L
Q57H
A701V
N501Y
E484K
K417N
22280-del
D215G
D80A
11287-del
K90R
K837N
T85I
C.
Delta mutations (amino acid)
D377Y
R203M
D63G
T120I
V82A
I82T
S26L
T77A
D950N
P681R
T478K
L452R
T19R
1
2
3
4
5
6
7
8
9
10
11
12
13
14
15
16
17
18
19
BCFtools
FreeBayes
GATK
iVar
LoFreq
VarScan
Frequency
25
50
75
100

Fig. S10

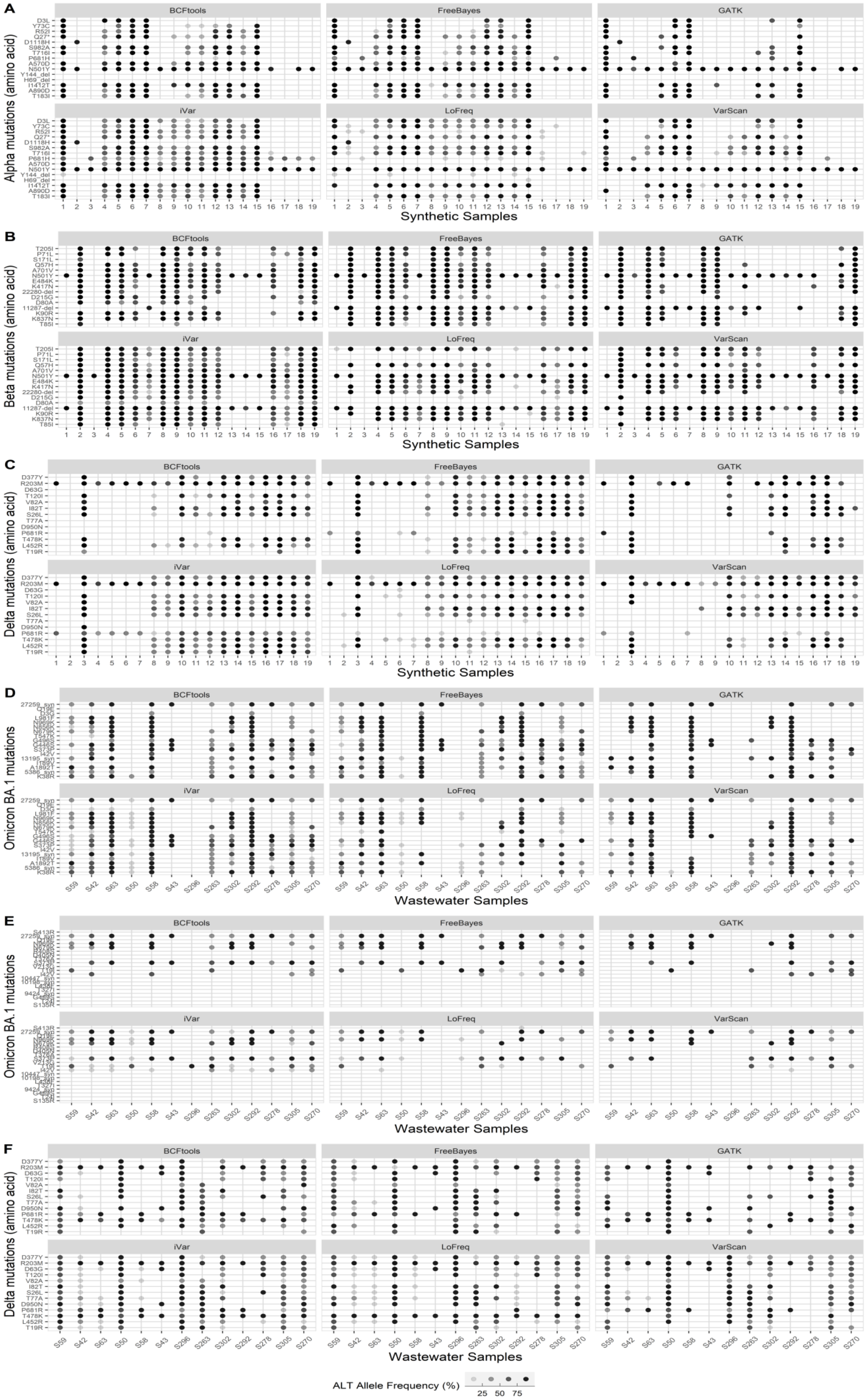
A
BCFtools
FreeBayes
GATK
iVar
LoFreq
VarScan
Alpha mutations (amino acid)
Synthetic Samples
B
Beta mutations (amino acid)
Synthetic Samples
C
Delta mutations (amino acid)
Synthetic Samples
D
Omicron BA.1 mutations
Wastewater Samples
E
Omicron BA.1 mutations
Wastewater Samples
F
Delta mutations (amino acid)
Wastewater Samples
ALT Allele Frequency (%)
25
50
75

Supplementary Figures 8-9-10

These figures represent the same outcome as described in Figure 6. They are meant to help interpret the results using different colouring options and facets given the complexity of the results described in the original plot.
