# Supplementary material for: Evaluation of variant calling algorithms for wastewater-based epidemiology using mixed populations of SARS-CoV-2 variants in synthetic and wastewater samples
Source: Microb Genom. 2023 Apr 19;9(4):mgen000933. doi: 10.1099/mgen.0.000933 (PMC10210938; doi:10.1099/mgen.0.000933)
Supplement: Supplementary material 5 [file mgen-9-933-s0011.pdf]

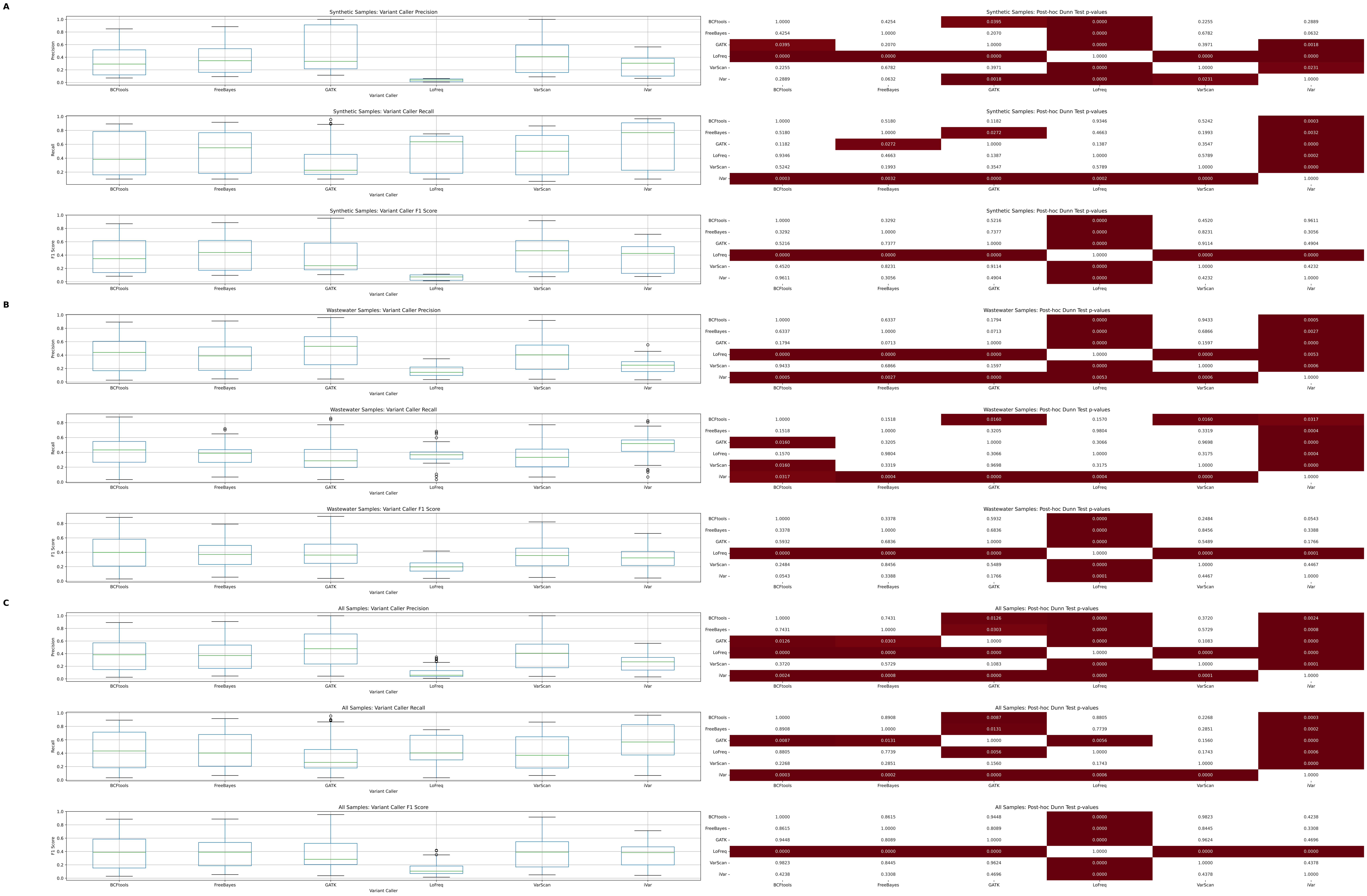

A
Synthetic Samples: Variant Caller Precision
Precision
Variant Caller
BCFtools FreeBayes GATK LoFreq VarScan iVar
Synthetic Samples: Post-hoc Dunn Test p-values
BCFtools 1.0000 0.4254 0.0395 0.0000 0.2255 0.2889
FreeBayes 0.4254 1.0000 0.2070 0.0000 0.6782 0.0632
GATK 0.0395 0.2070 1.0000 0.0000 0.3971 0.0018
LoFreq 0.0000 0.0000 0.0000 1.0000 0.0000 0.0000
VarScan 0.2255 0.6782 0.3971 0.0000 1.0000 0.0231
iVar 0.2889 0.0632 0.0018 0.0000 0.0231 1.0000
Synthetic Samples: Variant Caller Recall
Recall
Synthetic Samples: Post-hoc Dunn Test p-values
BCFtools 1.0000 0.5180 0.1182 0.9346 0.5242 0.0003
FreeBayes 0.5180 1.0000 0.0272 0.4663 0.1993 0.0032
GATK 0.1182 0.0272 1.0000 0.1387 0.3547 0.0000
LoFreq 0.9346 0.4663 0.1387 1.0000 0.5789 0.0002
VarScan 0.5242 0.1993 0.3547 0.5789 1.0000 0.0000
iVar 0.0003 0.0032 0.0000 0.0002 0.0000 1.0000
Synthetic Samples: Variant Caller F1 Score
F1 Score
Synthetic Samples: Post-hoc Dunn Test p-values
BCFtools 1.0000 0.3292 0.5216 0.0000 0.4520 0.9611
FreeBayes 0.3292 1.0000 0.7377 0.0000 0.8231 0.3056
GATK 0.5216 0.7377 1.0000 0.0000 0.9114 0.4904
LoFreq 0.0000 0.0000 0.0000 1.0000 0.0000 0.0000
VarScan 0.4520 0.8231 0.9114 0.0000 1.0000 0.4232
iVar 0.9611 0.3056 0.4904 0.0000 0.4232 1.0000
B
Wastewater Samples: Variant Caller Precision
Wastewater Samples: Post-hoc Dunn Test p-values
BCFtools 1.0000 0.6337 0.1794 0.0000 0.9433 0.0005
FreeBayes 0.6337 1.0000 0.0713 0.0000 0.6866 0.0027
GATK 0.1794 0.0713 1.0000 0.0000 0.1597 0.0000
LoFreq 0.0000 0.0000 0.0000 1.0000 0.0000 0.0053
VarScan 0.9433 0.6866 0.1597 0.0000 1.0000 0.0006
iVar 0.0005 0.0027 0.0000 0.0053 0.0006 1.0000
Wastewater Samples: Variant Caller Recall
Wastewater Samples: Post-hoc Dunn Test p-values
BCFtools 1.0000 0.1518 0.0160 0.1570 0.0160 0.0317
FreeBayes 0.1518 1.0000 0.3205 0.9804 0.3319 0.0004
GATK 0.0160 0.3205 1.0000 0.3066 0.9698 0.0000
LoFreq 0.1570 0.9804 0.3066 1.0000 0.3175 0.0004
VarScan 0.0160 0.3319 0.9698 0.3175 1.0000 0.0000
iVar 0.0317 0.0004 0.0000 0.0004 0.0000 1.0000
Wastewater Samples: Variant Caller F1 Score
Wastewater Samples: Post-hoc Dunn Test p-values
BCFtools 1.0000 0.3378 0.5932 0.0000 0.2484 0.0543
FreeBayes 0.3378 1.0000 0.6836 0.0000 0.8456 0.3388
GATK 0.5932 0.6836 1.0000 0.0000 0.5489 0.1766
LoFreq 0.0000 0.0000 0.0000 1.0000 0.0000 0.0001
VarScan 0.2484 0.8456 0.5489 0.0000 1.0000 0.4467
iVar 0.0543 0.3388 0.1766 0.0001 0.4467 1.0000
C
All Samples: Variant Caller Precision
All Samples: Post-hoc Dunn Test p-values
BCFtools 1.0000 0.7431 0.0126 0.0000 0.3720 0.0024
FreeBayes 0.7431 1.0000 0.0303 0.0000 0.5729 0.0008
GATK 0.0126 0.0303 1.0000 0.0000 0.1083 0.0000
LoFreq 0.0000 0.0000 0.0000 1.0000 0.0000 0.0000
VarScan 0.3720 0.5729 0.1083 0.0000 1.0000 0.0001
iVar 0.0024 0.0008 0.0000 0.0000 0.0001 1.0000
All Samples: Variant Caller Recall
All Samples: Post-hoc Dunn Test p-values
BCFtools 1.0000 0.8908 0.0087 0.8805 0.2268 0.0003
FreeBayes 0.8908 1.0000 0.0131 0.7739 0.2851 0.0002
GATK 0.0087 0.0131 1.0000 0.0056 0.1560 0.0000
LoFreq 0.8805 0.7739 0.0056 1.0000 0.1743 0.0006
VarScan 0.2268 0.2851 0.1560 0.1743 1.0000 0.0000
iVar 0.0003 0.0002 0.0000 0.0006 0.0000 1.0000
All Samples: Variant Caller F1 Score
All Samples: Post-hoc Dunn Test p-values
BCFtools 1.0000 0.8615 0.9448 0.0000 0.9823 0.4238
FreeBayes 0.8615 1.0000 0.8089 0.0000 0.8445 0.3308
GATK 0.9448 0.8089 1.0000 0.0000 0.9624 0.4696
LoFreq 0.0000 0.0000 0.0000 1.0000 0.0000 0.0000
VarScan 0.9823 0.8445 0.9624 0.0000 1.0000 0.4378
iVar 0.4238 0.3308 0.4696 0.0000 0.4378 1.0000
